# Supplementary material for: High prevalence of an alpha variant lineage with a premature stop codon in ORF7a in Iraq, winter 2020–2021
Source: PLoS One. 2022 May 26;17(5):e0267295. doi: 10.1371/journal.pone.0267295 (PMC9135184; doi:10.1371/journal.pone.0267295)
Supplement: S1 Table — (DOCX) [file pone.0267295.s001.docx]

We gratefully acknowledge the following Authors from the Originating laboratories responsible for obtaining the specimens and the

Submitting laboratories where genetic sequence data were generated and shared via the GISAID Initiative, on which this research is based.

| **Authors** | **Submitting laboratory** | **Originating laboratory** | **Collected** | **Accession No.** | **Virus name** |
| --- | --- | --- | --- | --- | --- |
| Peng Zhou, Xing-Lou Yang, Ding-Yu Zhang, Lei Zhang, Yan Zhu, Hao-Rui Si, Zhengli Shi | Wuhan Institute of Virology, Chinese Academy of Sciences | Wuhan Jinyintan Hospital | 2019-12-30 | EPI_ISL_402124 | **hCoV-19/Wuhan/WIV04/2019** |
| Nihad Al-Rashedi, Hussein Alburkat, Murad Munahi, Alaa Hameed, Ali Jasim, Olli Vapalahti, Tarja Sironen,Teemu Smura | Department of Virology, Faculty of Medicine, University of Helsinki, Helsinki, Finlan | Biology Department, College of Science, Al Muthanna University and Public Health Laboratory, Al-Muthanna Health Directorate | 2021-01-20 | EPI_ISL_1524330 | **hCoV-19/Iraq/Samawa-1/2021** |
| Nihad Al-Rashedi, Hussein Alburkat, Murad Munahi, Alaa Hameed, Ali Jasim, Olli Vapalahti, Tarja Sironen,Teemu Smura | Department of Virology, Faculty of Medicine, University of Helsinki, Helsinki, Finlan | Biology Department, College of Science, Al Muthanna University and Public Health Laboratory, Al-Muthanna Health Directorate | 2021-02-20 | EPI_ISL_1524331 | **hCoV-19/Iraq/Samawa-2/2021** |
| Nihad Al-Rashedi, Hussein Alburkat, Murad Munahi, Alaa Hameed, Ali Jasim, Olli Vapalahti, Tarja Sironen,Teemu Smura | Department of Virology, Faculty of Medicine, University of Helsinki, Helsinki, Finlan | Biology Department, College of Science, Al Muthanna University and Public Health Laboratory, Al-Muthanna Health Directorate | 2020-12-27 | EPI_ISL_1524332 | **hCoV-19/Iraq/Samawa-3/2021** |
| Nihad Al-Rashedi, Hussein Alburkat, Murad Munahi, Alaa Hameed, Ali Jasim, Olli Vapalahti, Tarja Sironen,Teemu Smura | Department of Virology, Faculty of Medicine, University of Helsinki, Helsinki, Finlan | Biology Department, College of Science, Al Muthanna University and Public Health Laboratory, Al-Muthanna Health Directorate | 2021-02-16 | EPI_ISL_1524333 | **hCoV-19/Iraq/Samawa-4/2021** |
| Nihad Al-Rashedi, Hussein Alburkat, Murad Munahi, Alaa Hameed, Ali Jasim, Olli Vapalahti, Tarja Sironen,Teemu Smura | Department of Virology, Faculty of Medicine, University of Helsinki, Helsinki, Finlan | Biology Department, College of Science, Al Muthanna University and Public Health Laboratory, Al-Muthanna Health Directorate | 2021-02-20 | EPI_ISL_1524334 | **hCoV-19/Iraq/Samawa-5/2021** |
| Nihad Al-Rashedi, Hussein Alburkat, Murad Munahi, Alaa Hameed, Ali Jasim, Olli Vapalahti, Tarja Sironen,Teemu Smura | Department of Virology, Faculty of Medicine, University of Helsinki, Helsinki, Finlan | Biology Department, College of Science, Al Muthanna University and Public Health Laboratory, Al-Muthanna Health Directorate | 2021-02-20 | EPI_ISL_1524335 | **hCoV-19/Iraq/Samawa-6/2021** |
| Nihad Al-Rashedi, Hussein Alburkat, Murad Munahi, Alaa Hameed, Ali Jasim, Olli Vapalahti, Tarja Sironen,Teemu Smura | Department of Virology, Faculty of Medicine, University of Helsinki, Helsinki, Finlan | Biology Department, College of Science, Al Muthanna University and Public Health Laboratory, Al-Muthanna Health Directorate | 2021-02-20 | EPI_ISL_1524336 | **hCoV-19/Iraq/Samawa-7/2021** |
| Nihad Al-Rashedi, Hussein Alburkat, Murad Munahi, Alaa Hameed, Ali Jasim, Olli Vapalahti, Tarja Sironen,Teemu Smura | Department of Virology, Faculty of Medicine, University of Helsinki, Helsinki, Finlan | Biology Department, College of Science, Al Muthanna University and Public Health Laboratory, Al-Muthanna Health Directorate | 2021-02-20 | EPI_ISL_1524337 | **hCoV-19/Iraq/Samawa-8/2021** |
| Nihad Al-Rashedi, Hussein Alburkat, Murad Munahi, Alaa Hameed, Ali Jasim, Olli Vapalahti, Tarja Sironen,Teemu Smura | Department of Virology, Faculty of Medicine, University of Helsinki, Helsinki, Finlan | Biology Department, College of Science, Al Muthanna University and Public Health Laboratory, Al-Muthanna Health Directorate | 2021-02-10 | EPI_ISL_1524338 | **hCoV-19/Iraq/Samawa-9/2021** |
| Nihad Al-Rashedi, Hussein Alburkat, Murad Munahi, Alaa Hameed, Ali Jasim, Olli Vapalahti, Tarja Sironen,Teemu Smura | Department of Virology, Faculty of Medicine, University of Helsinki, Helsinki, Finlan | Biology Department, College of Science, Al Muthanna University and Public Health Laboratory, Al-Muthanna Health Directorate | 2021-02-20 | EPI_ISL_1524339 | **hCoV-19/Iraq/Samawa-10/2021** |
| Nihad Al-Rashedi, Hussein Alburkat, Murad Munahi, Alaa Hameed, Ali Jasim, Olli Vapalahti, Tarja Sironen,Teemu Smura | Department of Virology, Faculty of Medicine, University of Helsinki, Helsinki, Finlan | Biology Department, College of Science, Al Muthanna University and Public Health Laboratory, Al-Muthanna Health Directorate | 2021-02-19 | EPI_ISL_1524340 | **hCoV-19/Iraq/Samawa-11/2021** |
| Nihad Al-Rashedi, Hussein Alburkat, Murad Munahi, Alaa Hameed, Ali Jasim, Olli Vapalahti, Tarja Sironen,Teemu Smura | Department of Virology, Faculty of Medicine, University of Helsinki, Helsinki, Finlan | Biology Department, College of Science, Al Muthanna University and Public Health Laboratory, Al-Muthanna Health Directorate | 2021-02-20 | EPI_ISL_1524341 | **hCoV-19/Iraq/Samawa-12/2021** |
| Nihad Al-Rashedi, Hussein Alburkat, Murad Munahi, Alaa Hameed, Ali Jasim, Olli Vapalahti, Tarja Sironen,Teemu Smura | Department of Virology, Faculty of Medicine, University of Helsinki, Helsinki, Finlan | Biology Department, College of Science, Al Muthanna University and Public Health Laboratory, Al-Muthanna Health Directorate | 2021-02-20 | EPI_ISL_1524342 | **hCoV-19/Iraq/Samawa-13/2021** |
| Nihad Al-Rashedi, Hussein Alburkat, Murad Munahi, Alaa Hameed, Ali Jasim, Olli Vapalahti, Tarja Sironen,Teemu Smura | Department of Virology, Faculty of Medicine, University of Helsinki, Helsinki, Finlan | Biology Department, College of Science, Al Muthanna University and Public Health Laboratory, Al-Muthanna Health Directorate | 2021-02-19 | EPI_ISL_1524343 | **hCoV-19/Iraq/Samawa-14/2021** |
| Nihad Al-Rashedi, Hussein Alburkat, Murad Munahi, Alaa Hameed, Ali Jasim, Olli Vapalahti, Tarja Sironen,Teemu Smura | Department of Virology, Faculty of Medicine, University of Helsinki, Helsinki, Finlan | Biology Department, College of Science, Al Muthanna University and Public Health Laboratory, Al-Muthanna Health Directorate | 2020-12-29 | EPI_ISL_1524344 | **hCoV-19/Iraq/Samawa-15/2021** |
| Nihad Al-Rashedi, Hussein Alburkat, Murad Munahi, Alaa Hameed, Ali Jasim, Olli Vapalahti, Tarja Sironen,Teemu Smura | Department of Virology, Faculty of Medicine, University of Helsinki, Helsinki, Finlan | Biology Department, College of Science, Al Muthanna University and Public Health Laboratory, Al-Muthanna Health Directorate | 2021-02-10 | EPI_ISL_1524345 | **hCoV-19/Iraq/Samawa-16/2021** |
| Nihad Al-Rashedi, Hussein Alburkat, Murad Munahi, Alaa Hameed, Ali Jasim, Olli Vapalahti, Tarja Sironen,Teemu Smura | Department of Virology, Faculty of Medicine, University of Helsinki, Helsinki, Finlan | Biology Department, College of Science, Al Muthanna University and Public Health Laboratory, Al-Muthanna Health Directorate | 2021-02-15 | EPI_ISL_1524346 | **hCoV-19/Iraq/Samawa-17/2021** |
| Nihad Al-Rashedi, Hussein Alburkat, Murad Munahi, Alaa Hameed, Ali Jasim, Olli Vapalahti, Tarja Sironen,Teemu Smura | Department of Virology, Faculty of Medicine, University of Helsinki, Helsinki, Finlan | Biology Department, College of Science, Al Muthanna University and Public Health Laboratory, Al-Muthanna Health Directorate | 2021-01-23 | EPI_ISL_1524347 | **hCoV-19/Iraq/Samawa-18/2021** |
| Nihad Al-Rashedi, Hussein Alburkat, Murad Munahi, Alaa Hameed, Ali Jasim, Olli Vapalahti, Tarja Sironen,Teemu Smura | Department of Virology, Faculty of Medicine, University of Helsinki, Helsinki, Finlan | Biology Department, College of Science, Al Muthanna University and Public Health Laboratory, Al-Muthanna Health Directorate | 2021-02-25 | EPI_ISL_1524348 | **hCoV-19/Iraq/Samawa-19/2021** |
| Nihad Al-Rashedi, Hussein Alburkat, Murad Munahi, Alaa Hameed, Ali Jasim, Olli Vapalahti, Tarja Sironen,Teemu Smura | Department of Virology, Faculty of Medicine, University of Helsinki, Helsinki, Finlan | Biology Department, College of Science, Al Muthanna University and Public Health Laboratory, Al-Muthanna Health Directorate | 2021-02-10 | EPI_ISL_1524349 | **hCoV-19/Iraq/Samawa-20/2021** |
| Nihad Al-Rashedi, Hussein Alburkat, Murad Munahi, Alaa Hameed, Ali Jasim, Olli Vapalahti, Tarja Sironen,Teemu Smura | Department of Virology, Faculty of Medicine, University of Helsinki, Helsinki, Finlan | Biology Department, College of Science, Al Muthanna University and Public Health Laboratory, Al-Muthanna Health Directorate | 2021-02-20 | EPI_ISL_1524350 | **hCoV-19/Iraq/Samawa-21/2021** |
| Nihad Al-Rashedi, Hussein Alburkat, Murad Munahi, Alaa Hameed, Ali Jasim, Olli Vapalahti, Tarja Sironen,Teemu Smura | Department of Virology, Faculty of Medicine, University of Helsinki, Helsinki, Finlan | Biology Department, College of Science, Al Muthanna University and Public Health Laboratory, Al-Muthanna Health Directorate | 2021-02-15 | EPI_ISL_1524351 | **hCoV-19/Iraq/Samawa-22/2021** |
| Nihad Al-Rashedi, Hussein Alburkat, Murad Munahi, Alaa Hameed, Ali Jasim, Olli Vapalahti, Tarja Sironen,Teemu Smura | Department of Virology, Faculty of Medicine, University of Helsinki, Helsinki, Finlan | Biology Department, College of Science, Al Muthanna University and Public Health Laboratory, Al-Muthanna Health Directorate | 2021-02-20 | EPI_ISL_1524352 | **hCoV-19/Iraq/Samawa-23/2021** |
| Nihad Al-Rashedi, Hussein Alburkat, Murad Munahi, Alaa Hameed, Ali Jasim, Olli Vapalahti, Tarja Sironen,Teemu Smura | Department of Virology, Faculty of Medicine, University of Helsinki, Helsinki, Finlan | Biology Department, College of Science, Al Muthanna University and Public Health Laboratory, Al-Muthanna Health Directorate | 2021-02-19 | EPI_ISL_1524353 | **hCoV-19/Iraq/Samawa-24/2021** |
| Nihad Al-Rashedi, Hussein Alburkat, Murad Munahi, Alaa Hameed, Ali Jasim, Olli Vapalahti, Tarja Sironen,Teemu Smura | Department of Virology, Faculty of Medicine, University of Helsinki, Helsinki, Finlan | Biology Department, College of Science, Al Muthanna University and Public Health Laboratory, Al-Muthanna Health Directorate | 2021-02-20 | EPI_ISL_1524354 | **hCoV-19/Iraq/Samawa-25/2021** |
| Nihad Al-Rashedi, Hussein Alburkat, Murad Munahi, Alaa Hameed, Ali Jasim, Olli Vapalahti, Tarja Sironen,Teemu Smura | Department of Virology, Faculty of Medicine, University of Helsinki, Helsinki, Finlan | Biology Department, College of Science, Al Muthanna University and Public Health Laboratory, Al-Muthanna Health Directorate | 2021-02-22 | EPI_ISL_1524355 | **hCoV-19/Iraq/Samawa-26/2021** |
| Nihad Al-Rashedi, Hussein Alburkat, Murad Munahi, Alaa Hameed, Ali Jasim, Olli Vapalahti, Tarja Sironen,Teemu Smura | Department of Virology, Faculty of Medicine, University of Helsinki, Helsinki, Finlan | Biology Department, College of Science, Al Muthanna University and Public Health Laboratory, Al-Muthanna Health Directorate | 2021-02-19 | EPI_ISL_1524356 | **hCoV-19/Iraq/Samawa-27/2021** |
| Nihad Al-Rashedi, Hussein Alburkat, Murad Munahi, Alaa Hameed, Ali Jasim, Olli Vapalahti, Tarja Sironen,Teemu Smura | Department of Virology, Faculty of Medicine, University of Helsinki, Helsinki, Finlan | Biology Department, College of Science, Al Muthanna University and Public Health Laboratory, Al-Muthanna Health Directorate | 2021-02-20 | EPI_ISL_1524357 | **hCoV-19/Iraq/Samawa-28/2021** |
| Nihad Al-Rashedi, Hussein Alburkat, Murad Munahi, Alaa Hameed, Ali Jasim, Olli Vapalahti, Tarja Sironen,Teemu Smura | Department of Virology, Faculty of Medicine, University of Helsinki, Helsinki, Finlan | Biology Department, College of Science, Al Muthanna University and Public Health Laboratory, Al-Muthanna Health Directorate | 2021-02-20 | EPI_ISL_1524358 | **hCoV-19/Iraq/Samawa-29/2021** |
| Nihad Al-Rashedi, Hussein Alburkat, Murad Munahi, Alaa Hameed, Ali Jasim, Olli Vapalahti, Tarja Sironen,Teemu Smura | Department of Virology, Faculty of Medicine, University of Helsinki, Helsinki, Finlan | Biology Department, College of Science, Al Muthanna University and Public Health Laboratory, Al-Muthanna Health Directorate | 2021-02-10 | EPI_ISL_1524359 | **hCoV-19/Iraq/Samawa-30/2021** |
| Nihad Al-Rashedi, Hussein Alburkat, Murad Munahi, Alaa Hameed, Ali Jasim, Olli Vapalahti, Tarja Sironen,Teemu Smura | Department of Virology, Faculty of Medicine, University of Helsinki, Helsinki, Finlan | Biology Department, College of Science, Al Muthanna University and Public Health Laboratory, Al-Muthanna Health Directorate | 2021-02-25 | EPI_ISL_1524360 | **hCoV-19/Iraq/Samawa-31/2021** |
| Nihad Al-Rashedi, Hussein Alburkat, Murad Munahi, Alaa Hameed, Ali Jasim, Olli Vapalahti, Tarja Sironen,Teemu Smura | Department of Virology, Faculty of Medicine, University of Helsinki, Helsinki, Finlan | Biology Department, College of Science, Al Muthanna University and Public Health Laboratory, Al-Muthanna Health Directorate | 2021-02-18 | EPI_ISL_1524361 | **hCoV-19/Iraq/Samawa-32/2021** |
| Nihad Al-Rashedi, Hussein Alburkat, Murad Munahi, Alaa Hameed, Ali Jasim, Olli Vapalahti, Tarja Sironen,Teemu Smura | Department of Virology, Faculty of Medicine, University of Helsinki, Helsinki, Finlan | Biology Department, College of Science, Al Muthanna University and Public Health Laboratory, Al-Muthanna Health Directorate | 2021-02-16 | EPI_ISL_1524362 | **hCoV-19/Iraq/Samawa-33/2021** |
| Nihad Al-Rashedi, Hussein Alburkat, Murad Munahi, Alaa Hameed, Ali Jasim, Olli Vapalahti, Tarja Sironen,Teemu Smura | Department of Virology, Faculty of Medicine, University of Helsinki, Helsinki, Finlan | Biology Department, College of Science, Al Muthanna University and Public Health Laboratory, Al-Muthanna Health Directorate | 2021-02-20 | EPI_ISL_1524363 | **hCoV-19/Iraq/Samawa-34/2021** |
| Nihad Al-Rashedi, Hussein Alburkat, Murad Munahi, Alaa Hameed, Ali Jasim, Olli Vapalahti, Tarja Sironen,Teemu Smura | Department of Virology, Faculty of Medicine, University of Helsinki, Helsinki, Finlan | Biology Department, College of Science, Al Muthanna University and Public Health Laboratory, Al-Muthanna Health Directorate | 2021-02-16 | EPI_ISL_1524364 | **hCoV-19/Iraq/Samawa-35/2021** |
| Nihad Al-Rashedi, Hussein Alburkat, Murad Munahi, Alaa Hameed, Ali Jasim, Olli Vapalahti, Tarja Sironen,Teemu Smura | Department of Virology, Faculty of Medicine, University of Helsinki, Helsinki, Finlan | Biology Department, College of Science, Al Muthanna University and Public Health Laboratory, Al-Muthanna Health Directorate | 2021-02-20 | EPI_ISL_1524365 | **hCoV-19/Iraq/Samawa-36/2021** |
| Nihad Al-Rashedi, Hussein Alburkat, Murad Munahi, Alaa Hameed, Ali Jasim, Olli Vapalahti, Tarja Sironen,Teemu Smura | Department of Virology, Faculty of Medicine, University of Helsinki, Helsinki, Finlan | Biology Department, College of Science, Al Muthanna University and Public Health Laboratory, Al-Muthanna Health Directorate | 2021-02-20 | EPI_ISL_1524366 | **hCoV-19/Iraq/Samawa-37/2021** |
| Nihad Al-Rashedi, Hussein Alburkat, Murad Munahi, Alaa Hameed, Ali Jasim, Olli Vapalahti, Tarja Sironen,Teemu Smura | Department of Virology, Faculty of Medicine, University of Helsinki, Helsinki, Finlan | Biology Department, College of Science, Al Muthanna University and Public Health Laboratory, Al-Muthanna Health Directorate | 2021-02-20 | EPI_ISL_1524367 | **hCoV-19/Iraq/Samawa-38/2021** |
| Nihad Al-Rashedi, Hussein Alburkat, Murad Munahi, Alaa Hameed, Ali Jasim, Olli Vapalahti, Tarja Sironen,Teemu Smura | Department of Virology, Faculty of Medicine, University of Helsinki, Helsinki, Finlan | Biology Department, College of Science, Al Muthanna University and Public Health Laboratory, Al-Muthanna Health Directorate | 2021-02-20 | EPI_ISL_1524368 | **hCoV-19/Iraq/Samawa-39/2021** |
| Nihad Al-Rashedi, Hussein Alburkat, Murad Munahi, Alaa Hameed, Ali Jasim, Olli Vapalahti, Tarja Sironen,Teemu Smura | Department of Virology, Faculty of Medicine, University of Helsinki, Helsinki, Finlan | Biology Department, College of Science, Al Muthanna University and Public Health Laboratory, Al-Muthanna Health Directorate | 2021-02-11 | EPI_ISL_1524369 | **hCoV-19/Iraq/Samawa-40/2021** |
| Nihad Al-Rashedi, Hussein Alburkat, Murad Munahi, Alaa Hameed, Ali Jasim, Olli Vapalahti, Tarja Sironen,Teemu Smura | Department of Virology, Faculty of Medicine, University of Helsinki, Helsinki, Finlan | Biology Department, College of Science, Al Muthanna University and Public Health Laboratory, Al-Muthanna Health Directorate | 2021-02-20 | EPI_ISL_1524370 | **hCoV-19/Iraq/Samawa-41/2021** |
| Nihad Al-Rashedi, Hussein Alburkat, Murad Munahi, Alaa Hameed, Ali Jasim, Olli Vapalahti, Tarja Sironen,Teemu Smura | Department of Virology, Faculty of Medicine, University of Helsinki, Helsinki, Finlan | Biology Department, College of Science, Al Muthanna University and Public Health Laboratory, Al-Muthanna Health Directorate | 2021-02-20 | EPI_ISL_1524371 | **hCoV-19/Iraq/Samawa-42/2021** |
| Nihad Al-Rashedi, Hussein Alburkat, Murad Munahi, Alaa Hameed, Ali Jasim, Olli Vapalahti, Tarja Sironen,Teemu Smura | Department of Virology, Faculty of Medicine, University of Helsinki, Helsinki, Finlan | Biology Department, College of Science, Al Muthanna University and Public Health Laboratory, Al-Muthanna Health Directorate | 2021-02-16 | EPI_ISL_1524372 | **hCoV-19/Iraq/Samawa-43/2021** |
| Nihad Al-Rashedi, Hussein Alburkat, Murad Munahi, Alaa Hameed, Ali Jasim, Olli Vapalahti, Tarja Sironen,Teemu Smura | Department of Virology, Faculty of Medicine, University of Helsinki, Helsinki, Finlan | Biology Department, College of Science, Al Muthanna University and Public Health Laboratory, Al-Muthanna Health Directorate | 2021-02-18 | EPI_ISL_1524373 | **hCoV-19/Iraq/Samawa-44/2021** |
| Nihad Al-Rashedi, Hussein Alburkat, Murad Munahi, Alaa Hameed, Ali Jasim, Olli Vapalahti, Tarja Sironen,Teemu Smura | Department of Virology, Faculty of Medicine, University of Helsinki, Helsinki, Finlan | Biology Department, College of Science, Al Muthanna University and Public Health Laboratory, Al-Muthanna Health Directorate | 2021-02-16 | EPI_ISL_1524374 | **hCoV-19/Iraq/Samawa-45/2021** |
| Nihad Al-Rashedi, Hussein Alburkat, Murad Munahi, Alaa Hameed, Ali Jasim, Olli Vapalahti, Tarja Sironen,Teemu Smura | Department of Virology, Faculty of Medicine, University of Helsinki, Helsinki, Finlan | Biology Department, College of Science, Al Muthanna University and Public Health Laboratory, Al-Muthanna Health Directorate | 2021-02-20 | EPI_ISL_1524375 | **hCoV-19/Iraq/Samawa-46/2021** |
| Nihad Al-Rashedi, Hussein Alburkat, Murad Munahi, Alaa Hameed, Ali Jasim, Olli Vapalahti, Tarja Sironen,Teemu Smura | Department of Virology, Faculty of Medicine, University of Helsinki, Helsinki, Finlan | Biology Department, College of Science, Al Muthanna University and Public Health Laboratory, Al-Muthanna Health Directorate | 2021-02-10 | EPI_ISL_1524376 | **hCoV-19/Iraq/Samawa-47/2021** |
| Nihad Al-Rashedi, Hussein Alburkat, Murad Munahi, Alaa Hameed, Ali Jasim, Olli Vapalahti, Tarja Sironen,Teemu Smura | Department of Virology, Faculty of Medicine, University of Helsinki, Helsinki, Finlan | Biology Department, College of Science, Al Muthanna University and Public Health Laboratory, Al-Muthanna Health Directorate | 2021-02-20 | EPI_ISL_1524377 | **hCoV-19/Iraq/Samawa-48/2021** |
| Nihad Al-Rashedi, Hussein Alburkat, Murad Munahi, Alaa Hameed, Ali Jasim, Olli Vapalahti, Tarja Sironen,Teemu Smura | Department of Virology, Faculty of Medicine, University of Helsinki, Helsinki, Finlan | Biology Department, College of Science, Al Muthanna University and Public Health Laboratory, Al-Muthanna Health Directorate | 2021-02-20 | EPI_ISL_1524378 | **hCoV-19/Iraq/Samawa-49/2021** |
| Nihad Al-Rashedi, Hussein Alburkat, Murad Munahi, Alaa Hameed, Ali Jasim, Olli Vapalahti, Tarja Sironen,Teemu Smura | Department of Virology, Faculty of Medicine, University of Helsinki, Helsinki, Finlan | Biology Department, College of Science, Al Muthanna University and Public Health Laboratory, Al-Muthanna Health Directorate | 2021-02-22 | EPI_ISL_1524379 | **hCoV-19/Iraq/Samawa-50/2021** |
| Nihad Al-Rashedi, Hussein Alburkat, Murad Munahi, Alaa Hameed, Ali Jasim, Olli Vapalahti, Tarja Sironen,Teemu Smura | Department of Virology, Faculty of Medicine, University of Helsinki, Helsinki, Finlan | Biology Department, College of Science, Al Muthanna University and Public Health Laboratory, Al-Muthanna Health Directorate | 2021-02-22 | EPI_ISL_1524380 | **hCoV-19/Iraq/Samawa-51/2021** |
| Nihad Al-Rashedi, Hussein Alburkat, Murad Munahi, Alaa Hameed, Ali Jasim, Olli Vapalahti, Tarja Sironen,Teemu Smura | Department of Virology, Faculty of Medicine, University of Helsinki, Helsinki, Finlan | Biology Department, College of Science, Al Muthanna University and Public Health Laboratory, Al-Muthanna Health Directorate | 2021-01-20 | EPI_ISL_1524381 | **hCoV-19/Iraq/Samawa-52/2021** |
| Nihad Al-Rashedi, Hussein Alburkat, Murad Munahi, Alaa Hameed, Ali Jasim, Olli Vapalahti, Tarja Sironen,Teemu Smura | Department of Virology, Faculty of Medicine, University of Helsinki, Helsinki, Finlan | Biology Department, College of Science, Al Muthanna University and Public Health Laboratory, Al-Muthanna Health Directorate | 2021-02-26 | EPI_ISL_2467906 | **hCoV-19/Iraq/Samawa-53/2021** |
| Nihad Al-Rashedi, Hussein Alburkat, Murad Munahi, Alaa Hameed, Ali Jasim, Olli Vapalahti, Tarja Sironen,Teemu Smura | Department of Virology, Faculty of Medicine, University of Helsinki, Helsinki, Finlan | Biology Department, College of Science, Al Muthanna University and Public Health Laboratory, Al-Muthanna Health Directorate | 2021-02-26 | EPI_ISL_2467907 | **hCoV-19/Iraq/Samawa-54/2021** |
| Nihad Al-Rashedi, Hussein Alburkat, Murad Munahi, Alaa Hameed, Ali Jasim, Olli Vapalahti, Tarja Sironen,Teemu Smura | Department of Virology, Faculty of Medicine, University of Helsinki, Helsinki, Finlan | Biology Department, College of Science, Al Muthanna University and Public Health Laboratory, Al-Muthanna Health Directorate | 2021-01-11 | EPI_ISL_2467908 | **hCoV-19/Iraq/Samawa-55/2021** |
| Nihad Al-Rashedi, Hussein Alburkat, Murad Munahi, Alaa Hameed, Ali Jasim, Olli Vapalahti, Tarja Sironen,Teemu Smura | Department of Virology, Faculty of Medicine, University of Helsinki, Helsinki, Finlan | Biology Department, College of Science, Al Muthanna University and Public Health Laboratory, Al-Muthanna Health Directorate | 2021-02-11 | EPI_ISL_2467909 | **hCoV-19/Iraq/Samawa-56/2021** |
| Nihad Al-Rashedi, Hussein Alburkat, Murad Munahi, Alaa Hameed, Ali Jasim, Olli Vapalahti, Tarja Sironen,Teemu Smura | Department of Virology, Faculty of Medicine, University of Helsinki, Helsinki, Finlan | Biology Department, College of Science, Al Muthanna University and Public Health Laboratory, Al-Muthanna Health Directorate | 2021-02-28 | EPI_ISL_2467910 | **hCoV-19/Iraq/Samawa-57/2021** |
| Nihad Al-Rashedi, Hussein Alburkat, Murad Munahi, Alaa Hameed, Ali Jasim, Olli Vapalahti, Tarja Sironen,Teemu Smura | Department of Virology, Faculty of Medicine, University of Helsinki, Helsinki, Finlan | Biology Department, College of Science, Al Muthanna University and Public Health Laboratory, Al-Muthanna Health Directorate | 2021-02-17 | EPI_ISL_2467911 | **hCoV-19/Iraq/Samawa-58/2021** |
| Nihad Al-Rashedi, Hussein Alburkat, Murad Munahi, Alaa Hameed, Ali Jasim, Olli Vapalahti, Tarja Sironen,Teemu Smura | Department of Virology, Faculty of Medicine, University of Helsinki, Helsinki, Finlan | Biology Department, College of Science, Al Muthanna University and Public Health Laboratory, Al-Muthanna Health Directorate | 2021-02-17 | EPI_ISL_2467912 | **hCoV-19/Iraq/Samawa-59/2021** |
| Nihad Al-Rashedi, Hussein Alburkat, Murad Munahi, Alaa Hameed, Ali Jasim, Olli Vapalahti, Tarja Sironen,Teemu Smura | Department of Virology, Faculty of Medicine, University of Helsinki, Helsinki, Finlan | Biology Department, College of Science, Al Muthanna University and Public Health Laboratory, Al-Muthanna Health Directorate | 2021-02-17 | EPI_ISL_2467913 | **hCoV-19/Iraq/Samawa-60/2021** |
| Nihad Al-Rashedi, Hussein Alburkat, Murad Munahi, Alaa Hameed, Ali Jasim, Olli Vapalahti, Tarja Sironen,Teemu Smura | Department of Virology, Faculty of Medicine, University of Helsinki, Helsinki, Finlan | Biology Department, College of Science, Al Muthanna University and Public Health Laboratory, Al-Muthanna Health Directorate | 2021-02-20 | EPI_ISL_2467914 | **hCoV-19/Iraq/Samawa-61/2021** |
| Nihad Al-Rashedi, Hussein Alburkat, Murad Munahi, Alaa Hameed, Ali Jasim, Olli Vapalahti, Tarja Sironen,Teemu Smura | Department of Virology, Faculty of Medicine, University of Helsinki, Helsinki, Finlan | Biology Department, College of Science, Al Muthanna University and Public Health Laboratory, Al-Muthanna Health Directorate | 2021-02-20 | EPI_ISL_2467915 | **hCoV-19/Iraq/Samawa-62/2021** |
| Nihad Al-Rashedi, Hussein Alburkat, Murad Munahi, Alaa Hameed, Ali Jasim, Olli Vapalahti, Tarja Sironen,Teemu Smura | Department of Virology, Faculty of Medicine, University of Helsinki, Helsinki, Finlan | Biology Department, College of Science, Al Muthanna University and Public Health Laboratory, Al-Muthanna Health Directorate | 2021-02-21 | EPI_ISL_2467916 | **hCoV-19/Iraq/Samawa-63/2021** |
| Nihad Al-Rashedi, Hussein Alburkat, Murad Munahi, Alaa Hameed, Ali Jasim, Olli Vapalahti, Tarja Sironen,Teemu Smura | Department of Virology, Faculty of Medicine, University of Helsinki, Helsinki, Finlan | Biology Department, College of Science, Al Muthanna University and Public Health Laboratory, Al-Muthanna Health Directorate | 2021-02-10 | EPI_ISL_2467917 | **hCoV-19/Iraq/Samawa-64/2021** |
| Nihad Al-Rashedi, Hussein Alburkat, Murad Munahi, Alaa Hameed, Ali Jasim, Olli Vapalahti, Tarja Sironen,Teemu Smura | Department of Virology, Faculty of Medicine, University of Helsinki, Helsinki, Finlan | Biology Department, College of Science, Al Muthanna University and Public Health Laboratory, Al-Muthanna Health Directorate | 2021-02-08 | EPI_ISL_2467918 | **hCoV-19/Iraq/Samawa-65/2021** |
| Nihad Al-Rashedi, Hussein Alburkat, Murad Munahi, Alaa Hameed, Ali Jasim, Olli Vapalahti, Tarja Sironen,Teemu Smura | Department of Virology, Faculty of Medicine, University of Helsinki, Helsinki, Finlan | Biology Department, College of Science, Al Muthanna University and Public Health Laboratory, Al-Muthanna Health Directorate | 2021-02-08 | EPI_ISL_2467919 | **hCoV-19/Iraq/Samawa-66/2021** |
| Nihad Al-Rashedi, Hussein Alburkat, Murad Munahi, Alaa Hameed, Ali Jasim, Olli Vapalahti, Tarja Sironen,Teemu Smura | Department of Virology, Faculty of Medicine, University of Helsinki, Helsinki, Finlan | Biology Department, College of Science, Al Muthanna University and Public Health Laboratory, Al-Muthanna Health Directorate | 2021-02-20 | EPI_ISL_2467920 | **hCoV-19/Iraq/Samawa-67/2021** |
| Nihad Al-Rashedi, Hussein Alburkat, Murad Munahi, Alaa Hameed, Ali Jasim, Olli Vapalahti, Tarja Sironen,Teemu Smura | Department of Virology, Faculty of Medicine, University of Helsinki, Helsinki, Finlan | Biology Department, College of Science, Al Muthanna University and Public Health Laboratory, Al-Muthanna Health Directorate | 2021-02-17 | EPI_ISL_2467921 | **hCoV-19/Iraq/Samawa-69/2021** |
| Nihad Al-Rashedi, Hussein Alburkat, Murad Munahi, Alaa Hameed, Ali Jasim, Olli Vapalahti, Tarja Sironen,Teemu Smura | Department of Virology, Faculty of Medicine, University of Helsinki, Helsinki, Finlan | Biology Department, College of Science, Al Muthanna University and Public Health Laboratory, Al-Muthanna Health Directorate | 2021-02-11 | EPI_ISL_2467922 | **hCoV-19/Iraq/Samawa-71/2021** |
| Nihad Al-Rashedi, Hussein Alburkat, Murad Munahi, Alaa Hameed, Ali Jasim, Olli Vapalahti, Tarja Sironen,Teemu Smura | Department of Virology, Faculty of Medicine, University of Helsinki, Helsinki, Finlan | Biology Department, College of Science, Al Muthanna University and Public Health Laboratory, Al-Muthanna Health Directorate | 2021-02-21 | EPI_ISL_2467923 | **hCoV-19/Iraq/Samawa-72/2021** |
| Nihad Al-Rashedi, Hussein Alburkat, Murad Munahi, Alaa Hameed, Ali Jasim, Olli Vapalahti, Tarja Sironen,Teemu Smura | Department of Virology, Faculty of Medicine, University of Helsinki, Helsinki, Finlan | Biology Department, College of Science, Al Muthanna University and Public Health Laboratory, Al-Muthanna Health Directorate | 2021-02-11 | EPI_ISL_2467924 | **hCoV-19/Iraq/Samawa-73/2021** |
| Nihad Al-Rashedi, Hussein Alburkat, Murad Munahi, Alaa Hameed, Ali Jasim, Olli Vapalahti, Tarja Sironen,Teemu Smura | Department of Virology, Faculty of Medicine, University of Helsinki, Helsinki, Finlan | Biology Department, College of Science, Al Muthanna University and Public Health Laboratory, Al-Muthanna Health Directorate | 2021-02-17 | EPI_ISL_2467925 | **hCoV-19/Iraq/Samawa-74/2021** |
| Nihad Al-Rashedi, Hussein Alburkat, Murad Munahi, Alaa Hameed, Ali Jasim, Olli Vapalahti, Tarja Sironen,Teemu Smura | Department of Virology, Faculty of Medicine, University of Helsinki, Helsinki, Finlan | Biology Department, College of Science, Al Muthanna University and Public Health Laboratory, Al-Muthanna Health Directorate | 2021-02-14 | EPI_ISL_2467926 | **hCoV-19/Iraq/Samawa-75/2021** |
| Nihad Al-Rashedi, Hussein Alburkat, Murad Munahi, Alaa Hameed, Ali Jasim, Olli Vapalahti, Tarja Sironen,Teemu Smura | Department of Virology, Faculty of Medicine, University of Helsinki, Helsinki, Finlan | Biology Department, College of Science, Al Muthanna University and Public Health Laboratory, Al-Muthanna Health Directorate | 2021-02-11 | EPI_ISL_2467927 | **hCoV-19/Iraq/Samawa-76/2021** |
| Nihad Al-Rashedi, Hussein Alburkat, Murad Munahi, Alaa Hameed, Ali Jasim, Olli Vapalahti, Tarja Sironen,Teemu Smura | Department of Virology, Faculty of Medicine, University of Helsinki, Helsinki, Finlan | Biology Department, College of Science, Al Muthanna University and Public Health Laboratory, Al-Muthanna Health Directorate | 2021-02-11 | EPI_ISL_2467928 | **hCoV-19/Iraq/Samawa-77/2021** |
| Nihad Al-Rashedi, Hussein Alburkat, Murad Munahi, Alaa Hameed, Ali Jasim, Olli Vapalahti, Tarja Sironen,Teemu Smura | Department of Virology, Faculty of Medicine, University of Helsinki, Helsinki, Finlan | Biology Department, College of Science, Al Muthanna University and Public Health Laboratory, Al-Muthanna Health Directorate | 2021-02-11 | EPI_ISL_2467929 | **hCoV-19/Iraq/Samawa-78/2021** |
| Samira Al-Maruqi, Fahad Zadjali, Amina Al Jardani, Khulood Al-Mammary, Hanan Al-kindi, Fatma BaAlawi, Hamida AL Barwani, Zeyana AL-Dahmani, Intisar Al-Shukri, Aisha Al-Busaidi, Aisha Al-Amri, Ahlam Al-Amri, Mohammed Al-Tobi, Samiha Al Kharusi, Abdulla Balkhair | Oman-NIC | Oman-NIC | 2020-03-21 | EPI_ISL_457990 | **hCoV-19/Oman/RESP-20-4400/2020** |
| Ndongo Dia, Moussa Moise Diagne, Mamadou Diop, Ousmane Faye, Amadou Alpha Sall  Submitter information | Institut Pasteur de Dakar | Institut Pasteur Dakar | 2020-03-17 | EPI_ISL_420069 | **hCoV-19/Senegal/306/2020** |
| Pablo Tsukayama, Alejandra Dávila-Barclay, Luis González, Pedro E. Romero, Brenda Ayzanoa, Janet Huancachoque, Pool Marcos, Maribel Huaringa | Laboratorio de Genómica Microbiana, Universidad Peruana Cayetano Heredia | Laboratorio de Referencia Nacional de Virus Respiratorios, Instituto Nacional de Salud Peru | 2020-06-08 | EPI_ISL_529067 | **hCoV-19/Peru/LAM-UPCH-0006/2020** |
| Ronnie Gavilan Chavez, Junior Caro Castro, Willi Quino Sifuentes, Veronica Hurtado Vela, Iris Silva Molina, Fiorella Orellana Peralta | Laboratorio de Referencia Nacional de Enteropatógenos. Instituto Nacional de Salud del Perú | Laboratorio de Referencia Nacional de Virus Respiratorio. Instituto Nacional de Salud Perú | 2020-03-16 | EPI_ISL_1111231 | **hCoV-19/Peru/LIM-INS-333/2020** |
| Siyuan Yang, Chengjie Jie, Fengting Yu, Yunxia Tang, Liting Yan, Linghang Wang | Laboratory of Infectious Diseases Center of Beijing Ditan Hospital | Laboratory of Infectious Diseases Center of Beijing Ditan Hospital | 2020-02-05 | EPI_ISL_452364 | **hCoV-19/Beijing/DT-BJ04/2020** |
| Nabaes Jodar, MS; Goya, S; Natale, MI; Lusso, S; Echavarría, M; Mistchenko, AS; Valinotto, LE; Viegas, M. | Área de Secuenciación del Laboratorio de Virología del Hospital de Niños Dr. Ricardo Gutierrez on behalf of 'Proyecto Argentino Interinstitucional de genomica de SARS-CoV-2' (PAIS Consortium) | Unidad de Virología, Centro de Educación Médica en Investigaciones Clínicas CEMIC | 2020-03-28 | EPI_ISL_792303 | **hCoV-19/Argentina/PAIS-A0222/2020** |
| Mélanie Albert, Marion Barbet, Sylvie Behillil, Méline Bizard, Angela Brisebarre, Flora Donati, Etienne Simon-Lorière, Vincent Enouf, Maud Vanpeene, Sylvie van der Werf, Fawzi Derrar | National Reference Center for Viruses of Respiratory Infections, Institut Pasteur, Paris | NIC Viral Respiratory Unit - Institut Pasteur of Algeria | 2020-06-09 | EPI_ISL_766869 | **hCoV-19/Algeria/G35014-8856/2020** |
| Rasmus Kirkegaard | Albertsen lab, Department of Chemistry and Bioscience, Aalborg University, Denmark | Department of Virus and Microbiological Special Diagnostics, Statens Serum Institut, Copenhagen, Denmark, Artillerivej 5, 2300 Copenahgen S | 2020-03-24 | EPI_ISL_429537 | **hCoV-19/Denmark/ALAB-SSI450/2020** |
| Joan Marti-Carreras, Bert Vanmechelen, Tony Wawina, Piet Maes | KU Leuven, Clinical and Epidemiological Virology | KU Leuven, Clinical and Epidemiological Virology | 2020-03-25 | EPI_ISL_420413 | **hCoV-19/Belgium/DJMG-0325156/2020** |
| Rasmus Kirkegaard | Albertsen lab, Department of Chemistry and Bioscience, Aalborg University, Denmark | Department of Clinical Microbiology, Copenhagen University Hospital, Hvidovre, Kettegaard Alle 30, 2650 Hvidovre | 2020-03-08 | EPI_ISL_451989 | **hCoV-19/Denmark/ALAB-HH-102/2020** |
| McHugh M, Dewar R, Rooke S, Gallagher M, Balcaza C, O'Toole A, Hill V, McCrone JT, Colquhoun R, Yu X, Jackson B, Scher E, Rambaut A, Williams TC, Templeton K | COVID-19 Genomics UK (COG-UK) Consortium | Virology Department, Royal Infirmary of Edinburgh, NHS Lothian / School of Biological Sciences, University of Edinburgh / Institute of Genetics and Molecular Medicine, University of Edinburgh | 2020-03-20 | EPI_ISL_425872 | **hCoV-19/Scotland/EDB075/2020** |
| Mélanie Albert, Marion Barbet, Sylvie Behillil, Méline Bizard, Angela Brisebarre, Flora Donati, Etienne Simon-Lorière, Vincent Enouf, Maud Vanpeene, Sylvie van der Werf, Fawzi Derrar | National Reference Center for Viruses of Respiratory Infections, Institut Pasteur, Paris | NIC Viral Respiratory Unit - Institut Pasteur of Algeria | 2020-06-15 | EPI_ISL_766863 | **hCoV-19/Algeria/G37318-8849/2020** |
| Vanessa G Allen, Philip Banh, Yao Chen, Richard de Borja, Alireza Eshaghi, Nahuel Fittipaldi, Christine Frantz, Jonathan B Gubbay, Jennifer L Guthrie, Lawrence Heisler, Esha Joshi, Michael Laszloffy, Aimin Li, Michael CY Li, Dean Maxwell, Sandeep Nagra, Samir N Patel, Jared Simpson, Karthikeyan Sivaraman, Ashleigh Sullivan, Yogi Sundaravadanam, Sarah Teatero, Andre Villegas, Matthew Watson, Sandra Zittermann | Public Health Ontario Laboratory | Public Health Ontario Laboratory | 2021-04 | EPI_ISL_2252203 | **hCoV-19/Canada/ON-PHL-21-16162/2021** |
| Dr. Varsha Potdar | NIV Influenza | ICMR-National Institute of Virology - INSACOG | 2021-02-11 | EPI_ISL_1703906 | **hCoV-19/India/GJ-ICMR-NIV-INSACOG-GSEQ-94/2021** |
| Drechsel, Oliver | Robert Koch Institute | SYNLAB MVZ Leverkusen | 2021-03-10 | EPI_ISL_1646809 | **hCoV-19/Germany/NW-RKI-I-081793/2021** |
| Baumeister E., Avaro M., Benedetti E., Russo M., Dattero ME, Pontoriero A., Cisterna D., Molina V., Perandones C., Tuduri E., Lorenzo F., Poklepovich T., Campos J. | Instituto Nacional Enfermedades Infecciosas C.G.Malbran | Servicio Virosis Respiratorias-Departamento Virología-INEI | 2021-02-04 | EPI_ISL_2135137 | **hCoV-19/Argentina/INEI101331/2021** |
| Issa Abu-Dayyeh, Ahmad Tibi, Lama Hussein, Shaima Ali, Badia Saddedin, Eiad Atwa, Amid Abdelnour | Biolab Diagnostic Laboratories | Biolab Diagnostic Laboratories | 2021-02-28 | \| EPI_ISL_2617455 \| \| --- \| | **hCoV-19/Jordan/Biolab0063/2021** |
| Khailany,R.A., Rahman,M.O., Ozaslan,M., Faraidun,H.N., Ibrahim,O.Q., Hama,H.A. and Kanabe,B.O. | Biology Department, Salahaddin University | Biology Department, Salahaddin University | 2021-03-09 | EPI_ISL_2629240 | **hCoV-19/Iraq/Erbil 5/2021** |
| Khailany,R.A., Rahman,M.O., Ozaslan,M., Faraidun,H.N., Ibrahim,O.Q., Hama,H.A. and Kanabe,B.O. | Biology Department, Salahaddin University | Biology Department, Salahaddin University | 2021-03-09 | EPI_ISL_2234383 | **hCoV-19-Iraq/Erbil 4/2021** |
| Lukas Endler, Anna Schedl, Fabian Amman, Petr Triska, Thomas Penz, Benedikt Agerer, Maelle Le Moing, Michael Schuster, Bekir Erguner, Jan Laine, Martin Senekowitsch, Christoph Bock, Andreas Bergthaler | Bergthaler laboratory, CeMM Research Center for Molecular Medicine of the Austrian Academy of Sciences | Department of Microbiology, University Innsbruck | 2021-02-05 | EPI_ISL_2232965 | **hCoV-19/env/ Liechtenstein/CeMM8894/2021** |
| Drechsel, Oliver | Robert Koch Institute | Bioscientia Labor Wermsdorf | 2021-02-15 | EPI_ISL_1155081 | **hCoV-19/Germany/SN-RKI-I-024907/2021** |
| Drechsel, Oliver | IMS-10036-CVDP-CAC2918B-8F48-491E-BD08-27ADDBE45777 | LADR MVZ Zweigpraxis Bernau | 2021-02-19 | EPI_ISL_1156444 | **hCoV-19/Germany/BB-RKI-I-026259/2021** |
| Roquebert B; Costa JM; Hedbaut E; Trombert S; Lecorche E; Verdurme L; Malek Ramdane, Olivi M; Haïm-Boukobza S. | CERBA LAB | CERBA | 2021-01-07 | EPI_ISL_862044 | **hCoV-19/France/IDF-CERBAHC-20211020-23/2021** |
| Marija Janevska, Hannelore Hamerlinck, Bruno Verhasselt | Lab voor klinische biologie | Lab voor klinische biologie | 2021-04-18 | EPI_ISL_2191955 | **hCoV-19/Belgium/UGent-5621/2021** |
| Antonio Grimaldi Patrizia Annunziata Francesco Panariello Teresa Giuliano Michele Cennamo Valentina Bouche Chiara Colantuono Lucio Di Filippo Mariano Fiorenza Anna Manfredi Marcello Salvi Giuseppe Portella Andrea Ballabio Davide Cacchiarelli | TIGEM | Università Federico II - Dipartimento di scienze mediche traslazionali - Napoli | 2021-02-01 | EPI_ISL_3014333 | **hCoV-19/Italy/CAM-TIGEM-IZSM-COLLI-17384/2021** |
| Radko Avi, Aare Abroi, Irja Lutsar, Kristi Huik, Taavi Päll, Meri Pauskar, Ene-Ly Jõgeda, Arina Shablinskaja, Eveli Kallas, Kai Truusalu, Dagmar Hoidmets, Katrin Kaarna, Tuuli Reisberg, Lili Azin Milani, Ulvi Gerst Talas, Heiki Niglas, Olga Sadikova, Liidia Dotsenko, Mari-Anne Härma, Kaisa Truus, Mats Hansen, Paul Naaber, Andrio Lahesaare | Department of Microbiology, Institute of Biomedicine and Translational Medicine, University of Tartu | SYNLAB Eesti OÜ | 2021-03-13 | EPI_ISL_2643420 | **hCoV-19/Estonia/Cov10119/2021** |
| anya Golubchik, David Bonsall, George Macintyre, Amy Trebes, Mariateresa de Cesare, Catrin Moore, Alex Mobbs, Anita Justice, Robert Shaw, Monique Andersson, Timothy Peto, Emma Wise, Nathan Moore, Jessica Lynch, Nick Cortes, Matilde Mori, Stephen Kidd, David Buck, John Todd, Christophe Fraser | COVID-19 Genomics UK (COG-UK) Consortium | Oxford Viromics, NDM, University of Oxford; Oxford University Hospitals; Basingstoke and North Hampshire Hospital | 2021-01-14 | EPI_ISL_999154 | **hCoV-19/England/OXON-F56F69/2021** |
| Kelvin K.W. To, Kwok-Yung Yuen | Department of Microbiology, The University of Hong Kong | Department of Microbiology, The University of Hong Kong | 2020-12-21 | EPI_ISL_1197085 | **hCoV-19/Hong Kong/HKU-210308-Imp4/2020** |
| Rossana Tallerico Federica Pasceri Marco De Fazio Ilenia Talotta Giuseppina Panduri Pasquale Minchella | Azienda Ospedaliera Pugliese Ciaccio di Catanzaro SOC Microbiologia e Virologia | Azienda Ospedaliera Pugliese Ciaccio di Catanzaro SOC Microbiologia e Virologia | 2021-05-01 | EPI_ISL_2282248 | **hCoV-19/Italy/CAL-AOCatanzaro-05011136/2021** |
| Daniel Ehrsam, Isabel StŸrmer, Catharine Aquino, Joel Wirz, Weihong Qi, Hubert Rehrauer, Verena Kufner, Gabriela Ziltener, Maryam Zaheri, Stefan Schmutz, Annette AudigŽ, Maria GrŸnberg, Kevin Steiner, Jon Huder, Cyril Shah, Riccarda Capaul, Guido Bloemberg, JŸrg Bšni, Michael Huber, Alexandra Trkola | Institute of Medical Virology | UniversitŠtsSpital ZŸrich | 2021-04-14 | EPI_ISL_1921818 | **hCoV-19/Switzerland/ZH-UZH-IMV-4555572d/2021** |
| Tanya Golubchik, David Bonsall, George Macintyre, Amy Trebes, Mariateresa de Cesare, Catrin Moore, Alex Mobbs, Anita Justice, Robert Shaw, Monique Andersson, Timothy Peto, Emma Wise, Nathan Moore, Jessica Lynch, Nick Cortes, Matilde Mori, Stephen Kidd, David Buck, John Todd, Christophe Fraser | COVID-19 Genomics UK (COG-UK) Consortium | Oxford Viromics, NDM, University of Oxford; Oxford University Hospitals; Basingstoke and North Hampshire Hospital | 2021-01-16 | EPI_ISL_999443 | **hCoV-19/England/OXON-F6044F/2021** |
| Lukas Endler, Anna Schedl, Fabian Amman, Petr Triska, Thomas Penz, Benedikt Agerer, Maelle Le Moing, Michael Schuster, Bekir Erguner, Jan Laine, Martin Senekowitsch, Christoph Bock, Andreas Bergthaler | Bergthaler laboratory, CeMM Research Center for Molecular Medicine of the Austrian Academy of Sciences | Department of Microbiology, University Innsbruck | 2021-04-25 | EPI_ISL_2137174 | **hCoV-19/env/Austria/CeMM8358/2021** |
| Laura Bankers, Molly C. Hetherington-Rauth, Diana Ir, Alexandria Rossheim, Shannon R. Matzinger, Sarah Elizabeth Totten, Emily A. Travanty | Colorado Department of Public Health and Environment | Colorado Department of Public Health and Environment | 2021-04-19 | EPI_ISL_2277317 | **hCoV-19/USA/CO-CDPHE-2100874634/2021** |
| Abigail C. Shockey, Alicia J. Mooney, Erika M. Hanson, Tonya Danz, Richard Griesser, Sara Wagner, Kelsey R. Florek | Wisconsin State Laboratory of Hygiene Communicable Disease Division | Wisconsin State Laboratory of Hygiene Communicable Disease Division | 2021-05-08 | EPI_ISL_2279344 | **hCoV-19/USA/WI-WSLH-214677/2021** |
| Placide Mbala-Kingebeni, Marie Claire Okomo, Edith Nkwembe, Eddy Kinganda-Lusamaki, Amuri Aziza, Francisca Muyembe Mawete, Emmanuel Lokilo Lofiko, Jean Claude Makangara, Raphael Lumembe, Gabriel Kabamba, Catherine Pratt, Matthias Pauthner, Josh Quick, Trevor Bedford, Ian Goodfellow, Andrew Rambaut, Nick Loman, Michael Wiley, Steve Ahuka-Mundeke, Jean-Jacques Muyembe Tamfum | Pathogen Genomics Lab, National Institute for Biomedical Research (INRB) | Laboratoire National de Santé Publique du Cameroun | 2021-03-03 | EPI_ISL_1972314 | **hCoV-19/Cameroon/CAM-31/2021** |
| Rashmi Tuladhar, Bonnie Oh, Jenny Zhang, Maliha Rahman, Mayela Pedrueza, Anita Pokharel, Karen Bobier, Lorraine Rodriguez, Myong Koag, Chun Wang, Rachel Lee, Grace Kubin | TXDSHS | TXDSHS | 2021-05-08 | EPI_ISL_2280052 | **hCoV-19/USA TX-DSHS-6592/2021** |
| Sadri,N., Alouani,D., Song,X | UHTL, University Hospitals | UHTL, University Hospitals | 2021-06-07 | EPI_ISL_2661529 | **hCoV-19/USA/OH-UHTL-992/2021** |
| Iglesias-Caballero, M. Sandonís,V. Vázquez-Morón, S. Camarero, S. Pozo, F. Casas, I. Jiménez, P. Zaballos, A. Monzón, S. Varona, S. Cuesta, I.SAINZ DE BARANDA CAMINO, CARIDAD | Instituto de Salud Carlos III | HOSPITAL GENERAL UNIVERSITARIO DE ALBACETE | 2021-03-31 | EPI_ISL_2331773 | **hCoV-19/Spain/CM-ISCIII-213286/2021** |
| Anastasia Chatzidimitriou et al. | Institute of Applied Biosciences, Centre for Research and Technology Hellas | Department of Microbiology, AHEPA University Hospital | 2021-05-05 | EPI_ISL_2235291 | **hCoV-19/Greece/V1183/2021** |
| PHE Covid Sequencing Team, Iris Hasibra, Prof Silvia Bino, Prof Albana Fico | Respiratory Virus Unit, National Infection Service, Public Health England | Unit of lab surveillance of viral emerging diseases, National Lab of Influenza | 2021-01-31 | EPI_ISL_1299858 | **hCoV-19/Albania/210901049/2021** |
| Jurre Y Siegers, Cecile Troupin, Leakhena Pum, Chiek Sivhour, Ly Sovann, Kraing Sidonn, Yi Sengdoeurn, Chin Savuth, Chau Darapheak, Veasna Duong, Erik A Karlsson | Virology Unit, Institut Pasteur du Cambodge | Battambang Provincial Laboratory | 2021-05-11 | EPI_ISL_2231569 | **hCoV-19/Cambodia/02-2105120711/2021** |
| Placide Mbala-Kingebeni, Edith Nkwembe, Eddy Kinganda-Lusamaki, Amuri Aziza, Francisca Muyembe Mawete, Emmanuel Lokilo Lofiko, Jean Claude Makangara, Catherine Pratt, Matthias Pauthner, Josh Quick, Allison Black, James Hadfield, Trevor Bedford, Ian Goodfellow, Andrew Rambaut, Nick Loman, Kristian Andersen, Michael Wiley, Steve Ahuka-Mundeke, Jean-Jacques Muyembe Tamfum | Pathogen Sequencing Lab, National Institute for Biomedical Research (INRB) | Viral Respiratory Lab, National Institute for Biomedical Research (INRB) | 2021-03-12 | EPI_ISL_2135839 | **hCoV-19/DRC/ 216884/2021** |
| Nada Madi, Hussain Safar, Ebaa Al-Awadhi, Anfal Al-Adwani | Virology Unit, Department of Microbiology, Faculty of Medicine, Kuwait University | Ministry of Health, Jaber Al-Ahmad Hospital | 2021-06-04 | EPI_ISL_2604034 | **hCoV-19/Kuwait/KU-96/2021** |
| Dominic N.C. Tsang, Daniel K.W. Chu, Haogao Gu, Tong Zhang, Leo L.M. Poon, Malik Peiris | School of Public Health, The University of Hong Kong | Hong Kong Department of Health | 2021-03 | EPI_ISL_1963623 | **hCoV-19/Hong Kong/CM21000226/2021** |
| Tim Roloff, Fanny Wegner, Helena MB Seth-Smith, Alfredo Mari, Karoline Leuzinger, Julia Bielicki, Christiane Beckmann, Manuel Battegay, Hans Hirsch, Adrian Egli | Clinical Bacteriology | Viollier AG | 2021-01-30 | EPI_ISL_2858611 | **hCoV-19/Switzerland/SO-UHB-001-31-551393-61/2021** |
| Lukas Endler, Anna Schedl, Fabian Amman, Petr Triska, Thomas Penz, Benedikt Agerer, Maelle Le Moing, Michael Schuster, Bekir Erguner, Jan Laine, Martin Senekowitsch, Christoph Bock, Andreas Bergthaler | Bergthaler laboratory, CeMM Research Center for Molecular Medicine of the Austrian Academy of Sciences | Austrian Agency for Health and Food Safety (AGES) | 2021-04-10 | EPI_ISL_1840940 | **hCoV-19/Austria/CeMM7670/2021** |
| Dr. Varsha Potdar | NIV Influenza | ICMR-National Institute of Virology - INSACOG | 2021-01-27 | EPI_ISL_1703962 | **hCoV-19/India/PB-ICMR-NIV-INSACOG- GSEQ-588/2021** |
| Alhamlan F,S., Al-Qahtani A,A., UdayaRaja G,K., Mutabagani M,S.,Balavenkatesh Mani,M., Althawadi S,I., Almaghrabi R,S., Alsanea M,S. and Alahideb B,M. | Infectious Diseases, King Faisal Hospital Research Cente | Infectious Diseases, King Faisal Hospital Research Center | 2021-04-15 | EPI_ISL_2151336 | **hCoV-19/Saudi Arabia/KFSHRC8/2021** |
| Endre Gábor Tóth, Balázs Somogyi, Ágnes Balázs-Nagy, Csaba Pereszlényi,Ferenc Jakab, Gábor Kemenesi | National Laboratory of Virology, Szentágothai Research Centre | Hungarian Defence Forces Military Medical Centre | 2021-01-22 | EPI_ISL_1041203 | **hCoV-19/Hungary/HM-0122-362/2021** |
| Sandra Janezic, Aleksander Mahnic, Maja Rupnik, Tjasa Žohar Čretnik, Alenka Štorman, Nika Gobec, Aleksander Kocuvan, Kaja Tominc, Maša Jarčič, David Cvetko, Tatjana Harlander, Matjaž Retelj / Jernej Kovač, Barbara Jenko Bizjan, Tine Tesovnik, Robert Šket, Katarina Kozmos, Ana Grom, Maruša Debeljak, Marko Pokorn, Tadej Battelino | NLZOH (National Laboratory for Health, Environment and Food) / CISLD (Clinical Institute of Special Laboratory Diagnostics), University Children's Hospital, University Medical Center Ljubljana | National Laboratory for Health, Environment and Food, OMM, Novo mesto | 2021-04-06 | EPI_ISL_1817500 | **hCoV-19/Slovenia/90-017770-NM/2021** |
| Arsen Arakelyan, Diana Avetyan, Siras Hakobyan, Gisane Khachatyan, Maria Nikoghosyan, Tamara Sirunyan, Nelli Muradyan, Andranik Chavushyan, Hovsep Ghazaryan, Roksana Zakharyan, Shushan Sargrsyan, Gayane Melik-Pashayan | Institute of Molecular Biology NAS RA, Republic of Armenia, Department of Bioengineering, BioinformaticsInstitute and Molecular Biology IBMPh RAU, Republic of Armenia | National Center of Disease Control and Prevention of the Republic of Armenia | 2021-03-18 | EPI_ISL_1718305 | **hCoV-19/Armenia/IMB3-12/2021** |
| Loai Alanagreh, Mustafa Ababneh, Abdel-Ellah Al-Shudifat , Mai Ajluny, Hanan Abu alshaikh, Foad Alzoughool, Mohammad-Borhan Al-zghoul, Manar Atoum | Alanagreh | Prince Hamzah Hospital | 2021-03 | EPI_ISL_1336799 | **hCoV-19/Jordan/AM-HU-13/2021** |
| Loai Alanagreh, Mustafa Ababneh, Abdel-Ellah Al-Shudifat , Mai Ajluny, Hanan Abu alshaikh, Foad Alzoughool, Mohammad-Borhan Al-zghoul, Manar Atoum | Alanagreh | Prince Hamzah Hospital | 2021-03 | EPI_ISL_1336798 | **hCoV-19/Jordan/AM-HU-6/2021** |
| Danish Covid-19 Genome Consortium | Aalborg University | Department of Virus and Microbiological Special Diagnostics, Statens Serum Institut, Copenhagen, Denmark | 2021-02-08 | EPI_ISL_1066476 | **hCoV-19/Denmark/DCGC-46965/2021** |
| Drechsel, Oliver | IMS-10182-CVDP-D16F47A3-44F0-47F4-9443-E9A6C8E7D099 | Labor Becker & Kollegen (Standort München) | 2021-02-23 | EPI_ISL_1433396 | **hCoV-19/Germany/BY-RKI-I-050934/2021** |
| Payel Mukherjee,Lamuk Zaveri,Tulasi Nagabandi, Ara Sreenivas,Shreekant Verma, Amareshwar Vodapalli ,Blessy B John,Viswagithe S L,B Himasri,Valli Nagalakshmi Undamatla,Onkar Kulkarni,Sofia Banu,Archana Bharadwaj Siva,Sharath Chandra Thota,Karthik Bharadwaj Tallapaka,Rakesh K Mishra,Divya Tej Sowpati | CSIR-Centre for Cellular and Molecular Biology-INSACOG | CSIR-Centre for Cellular and Molecular Biology | 2021-03-10 | EPI_ISL_1838367 | **hCoV-19/India/AP-CCMB-BJ395/2021** |
| Drechsel, Oliver | Robert Koch Institute | SYNLAB MVZ Neustadt a. d. Weinstraße | 2021-03-10 | EPI_ISL_1440628 | **hCoV-19/Germany/RP-RKI-I-056925/2021** |
| Catherine Moore, Johnathan Evans, Laura Gifford, Malorie Perry, Simon Cottrell, Angela Marchbank, Alec Birchley, Alexander Adams, Amy Gaskin, Bree Gatica-Wilcox, Jason Coombes, Joel Southgate, Lauren Gilbert, Lee Graham, Nicole Pacchiarini, Sara Kumziene-Summerhayes, Sarah Taylor, Sophie Jones, Sara Rey, Matthew Bull, Joanne Watkins, Sally Corden, Tom Connor | Public Health Wales Microbiology Cardiff Wales Specialist Virology Centre | Originating lab: Wales Specialist Virology Centre Sequencing lab: Pathogen Genomics Unit | 2021-01-08 | EPI_ISL_1476527 | **hCoV-19/Wales/PHWC-PYYW9U/2021** |
| Harper VanSteenhouse, Yumi Kasai, David Gray, Carol Clugston, Anna Dominiczak and Alex Alderton, Roberto Amato, Jeffrey Barrett, Sonia Goncalves, Ewan Harrison, David K. Jackson, Ian Johnston, Dominic Kwiatkowski, Cordelia Langford, John Sillitoe on behalf of the Wellcome Sanger Institute COVID-19 Surveillance Team | Wellcome Sanger Institute for the COVID-19 Genomics UK (COG-UK) Consortium | Lighthouse Lab in Glasgow | 2021-02-21 | EPI_ISL_1256517 | **hCoV-19/England/QEUH-12FDA6D/2021** |
| anya Golubchik, David Bonsall, George Macintyre, Amy Trebes, Mariateresa de Cesare, Catrin Moore, Alex Mobbs, Anita Justice, Robert Shaw, Monique Andersson, Timothy Peto, Emma Wise, Nathan Moore, Jessica Lynch, Nick Cortes, Matilde Mori, Stephen Kidd, David Buck, John Todd, Christophe Fraser | COVID-19 Genomics UK (COG-UK) Consortium | Oxford Viromics, NDM, University of Oxford; Oxford University Hospitals; Basingstoke and North Hampshire Hospital | 2020-12-28 | EPI_ISL_951006 | **hCoV-19/England/OXON-F481CE/2020** |
| Tanya Golubchik, David Bonsall, George Macintyre, Amy Trebes, Mariateresa de Cesare, Catrin Moore, Alex Mobbs, Anita Justice, Robert Shaw, Monique Andersson, Timothy Peto, Emma Wise, Nathan Moore, Jessica Lynch, Nick Cortes, Matilde Mori, Stephen Kidd, David Buck, John Todd, Christophe Fraser | COVID-19 Genomics UK (COG-UK) Consortium | Oxford Viromics, NDM, University of Oxford; Oxford University Hospitals; Basingstoke and North Hampshire Hospital | 2020-12-02 | EPI_ISL_1833046 | **hCoV-19/Scotland/OXON-F403B1/2020** |
| Fernando Lázaro, Rubén Cáceres, Jesús Mingorance Cruz, Elie Dahdouh | Servicio Microbiología Hospital La Paz | Servicio Microbiología Hospital La Paz | 2021-05-04 | EPI_ISL_2179789 | **hCoV-19/Spain/MD-59669058/2021** |
| Gilman Kit-Hang Siu, Lam-Kwong Lee, Kenneth Siu-Sing Leung, Jake Siu-Lun Leung, Timothy Ting-Leung Ng, Chloe Toi-Mei Chan, Kingsley King-Gee Tam, Hiu-Yin Lao, Denise Sze-Hang Wong, Alan Ka-Lun Wu, Miranda Chong-Yee Yau, Yvette Wai-Man Lai, Kitty Sau-Chun Fung, Sandy Ka-Yee Chau, Barry Kin-Chung Wong, Wing-Kin To,  Kristine Luk, Alex Yat-Man Ho, Tak-Lun Que, Kam-Tong Yip, Wing Cheong Yam, David Ho-Keung Shum, Shea Ping Yip | Department of Health Technology and Informatics, The Hong Kong Polytechnic University | Department of Health Technology and Informatics, The Hong Kong Polytechnic University | 2020-12-06 | EPI_ISL_1159106 | **hCoV-19/Hong Kong/HKPU-06804/2020** |
| Teemu Smura, Ravi Kant, Phuoc Truong, Hussein Alburkat, Hannimari Kallio-Kokko, Jenni Virtanen, Maija Suvanto, Essi Korhonen, Sari Hannula, Harri Kangas, Hanna Liimatainen, Satu Kurkela, Hanna Jarva, Maija Lappalainen, Pekka Ellonen, Olli Vapalaht | Department of Virology, Faculty of Medicine, University of Helsinki, Helsinki, Finland | Department of Virology and Immunology, University of Helsinki and Helsinki University Hospital, Huslab Finland | 2021-02-22 | EPI_ISL_2031006 | **hCoV-19/Finland/3228/2021** |
| Radko Avi, Aare Abroi, Irja Lutsar, Kristi Huik, Taavi Päll, Meri Pauskar, Ene-Ly Jõgeda, Arina Shablinskaja, Eveli Kallas, Kai Truusalu, Dagmar Hoidmets, Katrin Kaarna, Tuuli Reisberg, Lili Azin Milani, Ulvi Gerst Talas, Heiki Niglas, Olga Sadikova, Liidia Dotsenko, Mari-Anne Härma, Kaisa Truus, Mats Hansen, Paul Naaber, Andrio Lahesaare | Department of Microbiology, Institute of Biomedicine and Translational Medicine, University of Tartu | SYNLAB Eesti OÜ | 2021-03-30 | EPI_ISL_2642071 | **hCoV-19/Estonia/Cov11254/2021** |
| Michael Carr, Gabriel Gonzalez, Jonathan Dean, Cillian F De Gascun | National Virus Reference Laboratory | National Virus Reference Laboratory | 2021-01-14 | EPI_ISL_909912 | **hCoV-19/Ireland/SO-NVRL-83IRL56730/2021** |
| Irena Tabain, Ivana Ferenčak | Croatian Institute of Public Health | Croatian Institute of Public Health | 2021-02-17 | EPI_ISL_1272054 | **hCoV-19/Croatia/561/2021** |
| Neta Zuckerman, Efrat Dahan Bucris, Michal Mandelboim, Dana Bar-Ilan, Oran Erster, Tzvia Mann, Omer Murik, David A. Zeevi, Assaf Rokney, Joseph Jaffe, Eva Nachum, Maya Davidovich Cohen, Ephraim Fass, Gal Zizelski Valenci, Mor Rubinstein, Efrat Rorman, Israel Nissan, Efrat Glick-Saar, Omri Nayshool, Gideon Rechavi, Ella Mendelson, Orna Mor | Israel National Consortium for SARS-CoV-2 sequencing | Israel Central Virology laboratory | 2021-01-13 | EPI_ISL_944546 | **hCoV-19/Israel/CVL-2393/2021** |
| Teemu Smura, Ravi Kant, Phuoc Truong, Hussein Alburkat, Hannimari Kallio-Kokko, Jenni Virtanen, Maija Suvanto, Essi Korhonen, Sari Hannula, Harri Kangas, Hanna Liimatainen, Satu Kurkela, Hanna Jarva, Maija Lappalainen, Pekka Ellonen, Olli Vapalahti | Department of Virology, Faculty of Medicine, University of Helsinki, Helsinki, Finland | Department of Virology and Immunology, University of Helsinki and Helsinki University Hospital, Huslab Finland | 2021-02-19 | EPI_ISL_1841818 | **hCoV-19/Finland/2737/2021** |
| Teemu Smura, Ravi Kant, Phuoc Truong, Hussein Alburkat, Hannimari Kallio-Kokko, Jenni Virtanen, Maija Suvanto, Essi Korhonen, Sari Hannula, Harri Kangas, Hanna Liimatainen, Satu Kurkela, Hanna Jarva, Maija Lappalainen, Pekka Ellonen, Olli Vapalahti | Department of Virology, Faculty of Medicine, University of Helsinki, Helsinki, Finland | Department of Virology and Immunology, University of Helsinki and Helsinki University Hospital, Huslab Finland | 2021-02-04 | EPI_ISL_1842531 | **hCoV-19/Finland/V1070/2021** |
| Zaed,A., Altaif,Z., Shehab,F., AlWasti,H. | National Influenza Center, Bahrain | National Influenza Center, Bahrain | 2020-06-23 | EPI_ISL_486887 | **hCoV-19/Bahrain/02/2020** |
| Rachel Boyle, SallyAnn Harbison, Olivia Stroeven, Xiaoyun Ren, Matt Storey, Nikki Freed, Muhammad Faisal, Jing Wang, Hermes Perez, Anja Werno, Antje van der Linden, Arlo Upton, Chris Mansell, David Hammer, Dragana Drinkovic, Gary McAuliffe, Hana Sofia Andersson, James Ussher, Jill Sherwood, Josh Freeman, Julia Howard, Juliet Elvy, Mary DeAlmeida, Matt Blakiston, Matthew Rogers, Max Bloomfield, Michael Addidle, Michelle Balm, Sally Roberts, Sarah Jefferies, Sharmini Muttaiyah, Susan Morpeth, Susan Taylor, Timothy Blackmore, Vani Sathyendran, Veronica Playle, Virginia Hope, Erasmus Smit, Lauren Jelly, Olin Silander, Joep de Ligt | Institute of Environmental Science and Research (ESR) | LabPLUS | 2021-02-19 | EPI_ISL_1082268 | **hCoV-19/New Zealand/21MV0094/2021** |
| Alexandra Popa, Benedikt Agerer, Henrique Colaco, Lukas Endler, Jakob-Wendelin Genger, Alexander Lercher, Mark Smyth, Thomas Penz, Michael Schuster, Judith Aberle, Stephan Aberle, Elisabeth Puchhammer-Stöckl, Christoph Bock, Andreas Bergthaler | Bergthaler laboratory, CeMM Research Center for Molecular Medicine of the Austrian Academy of Sciences | Center for Virology, Medical University of Vienna | 2020-03-19 | EPI_ISL_419671 | **hCoV-19/Austria/CeMM0018/2020** |
| Pablo Tsukayama, Alejandra Dávila-Barclay, Luis González, Pedro E. Romero, Brenda Ayzanoa, Janet Huancachoque, Pool Marcos, Stella Chenet, Rafael Tapia, Cecilia Pajuelo, Carla Montenegro | Laboratorio de Genómica Microbiana, Universidad Peruana Cayetano Heredia | Instituto de Medicina Tropical, Universidad Nacional Toribio Rodríguez de Mendoza de Amazonas | 2020-09-28 | EPI_ISL_729907 | **hCoV-19/Peru/AMA-UPCH-0261/2020** |
| CIDM-PH et al. | NSW Health Pathology - Institute of Clinical Pathology and Medical Research; Westmead Hospital; University of Sydney | Sydney South West Pathology Service (SSWPS) - Royal Prince Alfred Hospital - NSW Health Pathology | 2021-01-08 | EPI_ISL_803111 | **hCoV-19/Australia/NSW1427/2021** |
| Rabeh El-Shesheny, Ahmed E Kayed, Ahmed El-Taweel, Mokhtar Gomaa, Sara Mahmoud, Yassmin Moatasim, Omnia Kutkat, Mina Kamel, Noura M Abo Shama, Mohamed El Sayes, Mahmoud Shehata, Ahmed Mostafa, Ahmed Kandeil, Richard Webby, Ghazi Kayali, Mohamed Ahmed Ali | Center of Scientific Excellence for Influenza Viruses (CSEIV), National Research Centre | Center of Scientific Excellence for Influenza Viruses (CSEIV), National Research Centre | 2020-08-17 | EPI_ISL_2232367 | **hCoV-19/Egypt/NRC-6465/2020** |
| Drechsel, Oliver | Robert Koch Institute | Bioscientia MVZ Labor Karlsruhe GmbH | 2021-02-08 | EPI_ISL_1148250 | **hCoV-19/Germany/BW-RKI-I-018088/2021** |
| Giandhari J, Pillay S, Lessells R, ChimukangaraB, Mdlalose K, York D, Khan S, Tegally H, Wilkinson E, de Oliveira T | KRISP, KZN Research Innovation and Sequencing Platform | MDS | 2020-12-07 | EPI_ISL_736927 | **hCoV-19/South Africa/KRISP-MDSH920916/2020** |
| Alhamlan F,S., Al-Qahtani A,A., Mutabagani M,S., Althawadi S,I.,Almaghrabi R,S., Alahideb B,M., Alsanea M,S., UdayaRaja G,K. and Balavenkatesh Mani,M. | BIOSCIENCE, King Faisal Hospital Research Center | BIOSCIENCE, King Faisal Hospital Research Center | 2021-04-15 | EPI_ISL_2001099 | **hCoV-19/Saudi Arabia/KFSHRC1/2021** |
| Caly L., Seemann T., Sait, M., Schultz M., Druce J., Sherry, N | Victorian Infectious Diseases Reference Laboratory and Microbiological Diagnostic Unit Public Health Laboratory, Doherty Institute | Victorian Infectious Diseases Reference Laboratory (VIDRL) | 2020-01-31 | EPI_ISL_419733 | **hCoV-19/Australia/VIC13/2020** |
| Saied Jaradat, Hazem Haddad, Areej Alquran, Maha Karam, Shereen Issa,Suha Hasan, Amid Abdelnour, Issa Abu-Dayyeh | Princess Haya Biotechnology Center/ Jordan University of Science & Technology | Biolab Diagnostic Laboratories | 2021-01-07 | EPI_ISL_935043 | **hCoV-19/Jordan/PHBC4/2021** |
| Bas Oude Munnink, David Nieuwenhuijse, Reina Sikkema, Fatema, Ebrahim Shehad, Amjad Ghanem Mohamed, Hashmeya Al Wasti, Claudia Schapendonk, Irina Chestakova, Anne van der Linden, Theo Bestebroer, Stefan van Nieuwkoop, Mark Pronk, Pascal Lexmond, Richard Molenkamp, Marion Koopmans, on behalf of the Dutch national COVID-19 response team. | Erasmus Medical Center | Kingdom of Bahrein Ministry of Health | 2020-03-30 | EPI_ISL_483553 | **hCoV-19/Bahrain/BAH-12/2020** |
| Xiaoyun Ren, Matt Storey, Nikki Freed, Muhammad Faisal, Jing Wang, Hermes Perez, Anja Werno, Antje van der Linden, Arlo Upton, Chris Mansell, David Hammer, Dragana Drinkovic, Gary McAuliffe, Hana Sofia Andersson, James Ussher, Jill Sherwood, Josh Freeman, Julia Howard, Juliet Elvy, Mary DeAlmeida, Matt Blakiston, Matthew Rogers, Max Bloomfield, | Institute of Environmental Science and Research (ESR) | Middlemore Hospital | 2020-03-23 | EPI_ISL_579303 | **hCoV-19/New Zealand/20VR2572/2020** |
| Andrés E. Castillo, Bárbara Parra, Paz Tapia, Alejandra Acevedo, Jaime Lagos, Winston Andrade, Loredana Arata, Gabriel Leal, Gisselle Barra, Carolina Tambley, Javier Tognarelli, Patricia Bustos, Soledad Ulloa, Rodrigo Fasce, Jorge Fernández. | Instituto de Salud Publica de Chile | Hospital de Talca, Chile | 2020-03-02 | EPI_ISL_414577 | **hCoV-19/Chile/ML-ISPCH-1/2020** |
| Xiaoyun Ren, Matt Storey, Nikki Freed, Muhammad Faisal, Jing Wang, Hermes Perez, Anja Werno, Antje van der Linden, Arlo Upton, Chris Mansell, David Hammer, Dragana Drinkovic, Gary McAuliffe, Hana Sofia Andersson, James Ussher, | Institute of Environmental Science and Research (ESR) | Canterbury Health Laboratories | 2020-03-23 | EPI_ISL_579446 | **hCoV-19/New Zealand/20VR3156/2020** |
| Kathrine Stene-Johansen, Kamilla Heddeland Instefjord, Hilde Elshaug, Garcia Llorente Ignacio, Jon Bråte, Engebretsen Serina Beate,Pedersen Benedikte Nevjen, Line Victoria Moen, Debech Nadia, Atiya R Ali,Marie Paulsen Madsen, Rasmus Riis Kopperud, Hilde Vollan, Karoline Bragstad, Olav Hungnes | Norwegian Institute of Public Health, Department of Virology | Oslo University Hospital, Department of Medical Microbiology | 2020-02-26 | EPI_ISL_2549107 | **hCoV-19/Norway/1378/2020** |
| Drechsel, Oliver | Robert Koch Institute | Eurofins LifeCodexx GmbH | 2021-03-06 | EPI_ISL_1432905 | **hCoV-19/Germany/BW-RKI-I-050580/2021** |
| Drechsel, Oliver | Robert Koch Institute | Eurofins LifeCodexx GmbH | 2021-03-05 | EPI_ISL_1640642 | **hCoV-19/Germany/BY-RKI-I-074312/2021** |
| Abu-Ali,H.M. and Al-Badran,I.F. | Department of Biology, University of Basrah | Department of Biology, University of Basrah | 2020-10-01 | EPI_ISL_907075 | **hCoV-19/Iraq BAS-2/2020** |
| PHE Covid Sequencing Team | Respiratory Virus Unit, Microbiology Services Colindale, Public Health England | Respiratory Virus Unit, Microbiology Services Colindale, Public Health England | 2020-02-27 | EPI_ISL_464200 | **hCoV-19/England/20099000504/2020** |
| Marion Barbet, Sylvie Behillil, Méline Bizard, Angela Brisebarre, Camille Capel, Etienne Simon-Lorière, Vincent Enouf, Maud Vanpeene, Sylvie van der Werf,Girard Sophie | National Reference Center for Viruses of Respiratory Infections, Institut Pasteur, Paris | Labo Analyses Med | 2021-01-03 | EPI_ISL_872282 | **hCoV-19/France/BFC-IPP00708/2021** |
| Consorcio Mexicano de Vigilancia Genómica (CoViGen-Mex). Authors (in alphabetical order): Julio Elias Alvarado-Yaah, Carlos F. Arias, Santiago Ávila-Ríos, Víctor Hugo Borja-Aburto, Celia Boukadida, Juan Bautista Chale-Dzul , José Antonio Enciso-Moreno, Gloria Elena Espinoza-Ayala, Fernando Fontove-Herrera, Concepción Grajales-Muñiz, Ricardo Grande, Alfredo Herrera-Estrella, | Instituto Nacional de Enfermedades Respiratorias (INER): Centro de Investigación en Enfermedades Infecciosas (CIENI) | Laboratorio Central de Epidemiología (LCE) | 2021-02-23 | EPI_ISL_1279455 | **hCoV-19/Mexico/CMX-INER-IMSS-00184/2021** |
| Rob Howes, The Lighthouse Lab in Cambridge and Alex Alderton, Roberto Amato, Sonia Goncalves, Ewan Harrison, David K. Jackson, Ian Johnston, Dominic Kwiatkowski, Cordelia Langford, John Sillitoe on behalf of the Wellcome Sanger Institute COVID-19 Surveillance Team | Wellcome Sanger Institute for the COVID-19 Genomics UK (COG-UK) Consortium | Lighthouse Lab in Cambridge | 2020-11-01 | EPI_ISL_647134 | **hCoV-19/England/CAMC-B218D9/2020** |
| Sharif Hala,Fadwa Alofi,Afrah Alsomali, Asim Khogeer, Sara Mfarrej, Khaled Algithami,Raeece Naeem, Amit Kumar Subudhi,Fathia Ben-Rached, Rahul Salunke, Anwar Hashem, Naif Almontashiri, Arnab Pain | Pathogen Genomics Lab King Abdullah University of Science and Technology(KAUST) | Pathogen Genomics Lab King Abdullah University of Science and Technology(KAUST) | 2020-03-30 | EPI_ISL_437751 | **hCoV-19/Saudi Arabia/KAUST-Madinah269/2020** |
| Fatma Bayrakdar, Yasemin Cosgun, Suleyman Yalcin, Gulay Korukluoglu | Ministry of Health Turkey | Ministry of Health Turkey | 2021-03-09 | EPI_ISL_1790199 | **hCoV-19/Turkey/HSGM-10992/2021** |
| Dakota Howard, Dhwani Batra, Peter W. Cook, Kara Moser, Adrian Paskey, Jason Caravas, Benjamin Rambo-Martin, Shatavia Morrison, Christopher Gulvick, Scott Sammons, Yvette Unoarumhi, Darlene Wagner, Matthew Schmerer, Eileen de Feo, Jan Antico, | Centers for Disease Control and Prevention Division of Viral Diseases, Pathogen Discovery | Helix/Illumina | 2021-04-09 | EPI_ISL_1796860 | **hCoV-19/USA/PA-CDC-STM-000052590/2021** |
| Neta Zuckerman, Efrat Dahan Bucris, Michal Mandelboim, Dana Bar-Ilan, Oran Erster, Tzvia Mann, Omer Murik, David A. Zeevi, Assaf Rokney, Joseph Jaffe, Eva Nachum, Maya Davidovich Cohen, Ephraim Fass, Gal Zizelski Valenci, Mor Rubinstein, Efrat Rorman, Israel Nissan, Efrat Glick-Saar, Omri Nayshool, Gideon Rechavi, Ella Mendelson, Orna Mor | Israel National Consortium for SARS-CoV-2 sequencing | Israel Central Virology laboratory | 2021-01-05 | EPI_ISL_1210225 | **hCoV-19/Israel/CVL-1461/2021** |
| Randall J. Olsen, Paul A. Christensen, S. Wesley Long, Sishir Subedi, Robert Olson, Marcus Nguyen, James J. Davis, Matthew Ojeda Saavedra, Prasanti Yerramilli, Layne Pruitt, Kristina Reppond, Madison N. Shyer, Jessica Cambric, Ryan Gadd, Ilya J. Finkelstein, Jimmy Gollihar, and James M. Musser | Houston Methodist Hospital | Houston Methodist Hospital | 2021-03-20 | EPI_ISL_2201206 | **hCoV-19/USA/TX-HMH-MCoV-35772/2021** |
| Son Nguyen | Public Health Virology-Forensic and Scientific Services (PHV-FSS) | Public Health Virology-Forensic and Scientific Services (PHV-FSS) | 2020-12-22 | EPI_ISL_1300530 | **hCoV-19/Australia/QLD1470/2020** |
| Marion Barbet, Sylvie Behillil, Méline Bizard, Angela Brisebarre, Camille Capel, Etienne Simon-Lorière, Vincent Enouf, Maud Vanpeene, Sylvie van der Werf,Gaudy Graffin Catherine | National Reference Center for Viruses of Respiratory Infections, Institut Pasteur, Paris | CHU Tours - Virologie | 2021-01-08 | EPI_ISL_860909 | **hCoV-19/France/CVL-IPP00317/2021** |
| Fatma Bayrakdar, Yasemin Cosgun, Suleyman Yalcin, Gulay Korukluoglu | Ministry of Health Turkey | Ministry of Health Turkey | 2021-03-11 | EPI_ISL_2157882 | **hCoV-19/Turkey/HSGM-11079/2021** |
| Abdulhussein Thair, Fadhil Hula | PCR lab | The teaching Baghdad hospital | 2021-02-26 | EPI_ISL_2379893 | **hCoV-19/Iraq/PCRLab-01/2021** |
| Alexandra Popa, Benedikt Agerer, Henrique Colaco, Lukas Endler, Jakob-Wendelin Genger, Alexander Lercher, Mark Smyth, Thomas Penz, Michael Schuster, Jan Laine, Martin Senekowitsch, Judith Aberle, Stephan Aberle, Peter Hufnagl, Daniela Schmid | Bergthaler laboratory, CeMM Research Center for Molecular Medicine of the Austrian Academy of Sciences | Austrian Agency for Health and Food Safety (AGES | 2020-07-19 | EPI_ISL_583885 | **hCoV-19/Austria/CeMM1083/2020** |
| Michael Carr, Gabriel Gonzalez, Jonathan Dean, Suzie Coughlan, Cillian F De Gascun | National Virus Reference Laboratory | National Virus Reference Laboratory | 2020-07-06 | EPI_ISL_500582 | **hCoV-19/Ireland/WD-NVRL-71IRL06472/2020** |
| Kathrine Stene-Johansen, Kamilla Heddeland Instefjord, Hilde Elshaug, Marie Paulsen Madsen, Rasmus Riis Kopperud, Hilde Vollan, Karoline Bragstad, Olav Hungnes | Norwegian Institute of Public Health, Department of Virology | University Hospital of Northern Norway, Department for Microbiology and Infectious Disease Control | 2020-07-31 | EPI_ISL_635186 | **hCoV-19/Norway/3584/2020** |
| Markus Antwerpen, Alexandra Rehn, Mathias Walter, Malena Bestehorn-Willmann, Sabine Zange, Enrico Georgi, Roman Wölfel | Bundeswehr Institute of Microbiology | Bundeswehr Institute of Microbiology | 2020-06-02 | EPI_ISL_1001000 | **hCoV-19/Afghanistan/IMB07962/2020** |
| Gabriela Moura, Sofia Marques, Patricia Arinto, Miguel Pinheiro and Manuel Santos | Institute of Biomedicine (IBiMED), Universidade de Aveiro | Centro Hospitalar de Entre o Douro e Vouga (CHEDV) | 2021-01-26 | EPI_ISL_1494779 | **hCoV-19/Portugal/PT5110/2021** |
| Danish Covid-19 Genome Consortium | Albertsen Lab, Department of Chemistry and Bioscience, Aalborg University, Denmark | Department of Virus and Microbiological Special Diagnostics, Statens Serum Institut, Copenhagen, Denmark | 2020-12-14 | EPI_ISL_751130 | **hCoV-19/Denmark/DCGC-17661/2020** |
| Jacquelyn Wynn, Mairead Hyland, The Lighthouse Lab in Alderley Park and Alex Alderton, Roberto Amato, Sonia Goncalves, Ewan Harrison, David K. Jackson, Ian Johnston, Dominic Kwiatkowski, Cordelia Langford, John Sillitoe on behalf of the Wellcome Sanger Institute COVID-19 Surveillance Team | Wellcome Sanger Institute for the COVID-19 Genomics UK (COG-UK) Consortium | Lighthouse Lab in Alderley Park | 2020-11-14 | EPI_ISL_675632 | **hCoV-19/England/ALDP-B83161/2020** |
| Nihad Al-Rashedi, Danilo Licastro, Sreejith Rajasekharan, Simeone Dal Monego, Alessandro Marcello | International Centre for Genetic Engineering and Biotechnology (ICGEB) and ARGO Open Lab Platform | Biology Department, College of Science, Al-Muthanna University | 2020-06-30 | EPI_ISL_582030 | **hCoV-19/Iraq/ICGEB-5T/2020** |
| Basil Britto Xavier, Jasmine Coppens, Marie Le Mercier, Christine Lammens, Veerle Matheeussen, Herman Goossens | UAntwerp, Laboratory of Medical Microbiology | Platform BIS UZA/UAntwerpen | 2021-02-24 | EPI_ISL_1190853 | **hCoV-19/Belgium/UZA-UA-CV2008146136/2021** |
| Issa Abu-Dayyeh, Ahmad Tibi, Lama Hussein, Shayma Ali, Badia Saddedin, Eiad Atwa, Amid Abdelnour | Biolab Diagnostic Laboratories | Biolab Diagnostic Laboratories | 2021-04-16 | EPI_ISL_1823202 | **hCoV-19/Jordan/Biolab020/2021** |
| Stacy Reeves, Jonathan Edwards, Cynthia Dixey, Tonia Parrott, Aliyah Fields, Taylor Smith | GA Department of Public Health | GA Department of Public Health | 2021-02-05 | EPI_ISL_1262463 | **hCoV-19/USA/GA-GPHL-0057/2021** |
| Xiaoyun Ren, Matt Storey, Nikki Freed, Muhammad Faisal, Jing Wang, Hermes Perez, Anja Werno, Antje van der Linden, Arlo Upton, Chris Mansell, David Hammer, Dragana Drinkovic, Gary McAuliffe, Hana Sofia Andersson, James Ussher, Jill Sherwood, Josh Freeman, Julia Howard, Juliet Elvy, Mary DeAlmeida, Matt Blakiston, Matthew Rogers, Max Bloomfield, Michael Addidle, Michelle Balm, Sally Roberts, Sarah Jefferies, Sharmini Muttaiyah, Susan Morpeth, Susan Taylor, Timothy Blackmore, Vani Sathyendran, Veronica Playle, Virginia Hope, Erasmus Smit, Lauren Jelly, Olin Silander, Joep de Ligt | Institute of Environmental Science and Research (ESR) | Middlemore Hospital | 2020-12-09 | EPI_ISL_732969 | **hCoV-19/New Zealand/20CV0669/2020** |
| Salman Al-Sabah , Mohammad Alghounaim | MOH - Jaber Al-Ahmad Hospital (Innovation Research Laboratory) | MOH - Jaber Al-Ahmad Hospital (Innovation Research Laboratory) | 2020-12-27 | EPI_ISL_903389 | **hCoV-19/Kuwait/SKS00103-JAH/2020** |
| Nihad Al-Rashedi, Danilo Licastro, Sreejith Rajasekharan, Simeone Dal Monego, Alessandro Marcello | International Centre for Genetic Engineering and Biotechnology (ICGEB) and ARGO Open Lab Platform | Biology Department, College of Science, Al-Muthanna University | 2020-06-30 | EPI_ISL_582029 | **hCoV-19/Iraq ICGEB-2T/2020** |
| Baillie Vicky, du Plessis Jeanine, Giandhari Jennifer, Pillay Sureshnee, Naidoo Yeshnee, Tegally Houriiyah, de Oliveira Tulio, Madhi Shabir | KRISP, KZN Research Innovation and Sequencing Platform | Vaccines and Infectious Diseases Analytics Research Unit (VIDA | 2020-11-12 | EPI_ISL_940877 | **hCoV-19/South Africa/VIDA-KRISP-V001019/2020** |
| Andrew Lang, Timelia Fink, Glen Gallagher, Sandra Smole | Massachusetts State Public Health Laboratory | Massachusetts State Public Health Laboratory | 2021-04-19 | EPI_ISL_2008374 | **hCoV-19/USA/MA-MASPHL-03369/2021** |
| Teemu Smura, Ravi Kant, Phuoc Truong, Hussein Alburkat, Hannimari Kallio-Kokko, Jenni Virtanen, Maija Suvanto, Essi Korhonen, Sari Hannula, Harri Kangas, Hanna Liimatainen, Satu Kurkela, Hanna Jarva, Maija Lappalainen, Pekka Ellonen, Olli Vapalahti | Department of Virology, Faculty of Medicine, University of Helsinki, Helsinki, Finland | Department of Virology and Immunology, University of Helsinki and Helsinki University Hospital, Huslab Finland | 2021-03-09 | EPI_ISL_2259002 | **hCoV-19/Finland/4992/2021** |
| AIT YAHYA Emilie, ALIDJINOU Enagnon Kazali, BOCKET Laurence, CREPIN Michel, DEMAY Christophe, ENGELMANN Ilka, GEFFROY Sandrine, GUIGON Aurélie, LAMBERT Valérie, LAZREK Mouna, NOBILLIAUX Florian, PREVOST Brigitte, THUILLIER Caroline, TINEZ Claire | CHU Lille - Laboratoire de Virologie | CH Lens | 2021-04-07 | EPI_ISL_1818807 | **hCoV-19/France/HDF-P037-21144M1410/2021** |
| Giandhari J, Pillay S, Lessells R, ChimukangaraB, Mdlalose K, York D, Khan S, Tegally H, Wilkinson E, de Oliveira T | KRISP, KZN Research Innovation and Sequencing Platform | NHLS-IALCH | 2020-11-25 | EPI_ISL_736954 | **hCoV-19/South Africa/KRISP-EG00467095/2020** |
| Alhamlan F,S., Al-Qahtani A,A., Mutabagani M,S., Althawadi S,I.,Almaghrabi R,S., Alahideb B,M., Alsanea M,S., Balavenkatesh Mani,M. and UdayaRaja G,K. | Infectious Diseases, King Faisal Hospital Research Center | Infectious Diseases, King Faisal Hospital Research Center | 2021-04-15 | EPI_ISL_2151337 | **hCoV-19/Saudi Arabia/KFSHRC10/2021** |
| Abu-Ali,H.F. and Al-Badran,A.I. | Department of Biology, University of Basrah | Department of Biology, University of Basrah | 2020-10-01 | EPI_ISL_956332 | **hCoV-19/Iraq/BAS-1/2020** |
| Anthony Fries, Jennifer Meyer, Amanda Javorina, Sarah Purves, William Gruner, Clarise Starr, Elizabeth Macias, Fritz Castillo, Cole Anderson | United States Air Force School of Aerospace Medicine | Landstuhl Regional Medical Center | 2020-06-17 | EPI_ISL_812285 | **hCoV-19/Iraq/USAFSAM-S092/2020** |
| Issa Abu-Dayyeh, Ahmad Tibi, Lama Hussein, Lina Mohammad, Zein Naber, Amid Abdelnour with SEARCH Alliance San Diego | Andersen lab at Scripps Research | Biolab Diagnostic Laboratories | 2020-03-28 | EPI_ISL_429998 | **hCoV-19/Jordan/SR-039/2020** |
| Xiaoyun Ren, Matt Storey, Nikki Freed, Muhammad Faisal, Jing Wang, Hermes Perez, Anja Werno, Antje van der Linden, Arlo Upton, Chris Mansell, David Hammer, Dragana Drinkovic, Gary McAuliffe, Hana Sofia Andersson, James Ussher, Jill Sherwood, Josh Freeman, Julia Howard, Juliet Elvy, Mary DeAlmeida, Matt Blakiston, Matthew Rogers, Max Bloomfield, Michael Addidle, Michelle Balm, Sally Roberts, Sarah Jefferies, Sharmini Muttaiyah, Susan Morpeth, Susan Taylor, Timothy Blackmore, Vani Sathyendran, Veronica Playle, Virginia Hope, Erasmus Smit, Lauren Jelly, Olin Silander, Joep de Ligt | Institute of Environmental Science and Research (ESR) | Canterbury Health Laboratories | 2020-03-27 | EPI_ISL_579081 | **hCoV-19/New Zealand/20CV0291/2020** |
| Sara Mfarrej, Olga Douvropoulou, Raushan Nugmanova, Raeece Naeem, Sharif Hala, Fadwa Alofi, Asim Khogeer, Afrah Alsomali, Jumana Taha, Abdulaziz Alahmadi, Kahled Algithami, Anwar Hashem, Naif Almontashiri, Arnab Pain | Pathogen Genomics Lab King Abdullah University of Science and Technology(KAUST) | Pathogen Genomics Lab King Abdullah University of Science and Technology(KAUST) | 2020-08-23 | EPI_ISL_751235 | **hCoV-19/Saudi Arabia/KAUST-MADINAH1655/2020** |
| Gilman Kit-Hang Siu, Lam-Kwong Lee, Kenneth Siu-Sing Leung, Jake Siu-Lun Leung, Timothy Ting-Leung Ng, Chloe Toi-Mei Chan, Kingsley King-Gee Tam, Hiu-Yin Lao, Denise Sze-Hang Wong, Alan Ka-Lun Wu, Miranda Chong-Yee Yau, Yvette Wai-Man Lai, Kitty Sau-Chun Fung, Sandy Ka-Yee Chau, Barry Kin-Chung Wong, Wing-Kin To,  Kristine Luk, Alex Yat-Man Ho, Tak-Lun Que, Kam-Tong Yip, Wing Cheong Yam, David Ho-Keung Shum, Shea Ping Yip | Department of Health Technology and Informatics, The Hong Kong Polytechnic University | Department of Health Technology and Informatics, The Hong Kong Polytechnic University | 2020-03-20 | EPI_ISL_1289436 | **hCoV-19/Hong Kong/HKPU-00248/2020** |
| Antonio Orduña-Domingo, Marta Hernandez, David Abad, Marta Dominguez-Gil, Silvia Rojo, Gabriel March Rossello, Sonsoles Garcinuño Pérez, Carmen Aldea-Mansilla, Mª Fe Brezmes-Valdivieso, Gregoria Megías Lobón, María Antonia García Castro, Carmen Gimeno Crespo, Noelia Arenal Andrés, Carlos Fuster Foz, M. Isabel Fernandez-Natal, Jose María Eiros Bouza | SARS-CoV-2 Sequencing Castilla y Leon-Spain Consortium | SARS-CoV-2 Sequencing Castilla y Leon-Spain Consortium | 2021-06-14 | EPI_ISL_2986426 | **hCoV-19/Spain/CL-COV05707/2021** |
| John K. Everett, Kyle Rodino, Shantan Reddy, Aoife M. Roche, Young Hwang, Scott Sherrill-Mix, Samantha A. Whiteside, Jevon Graham-Wooten, Layla A. Khatib, Ayannah S. Fitzgerald, Arupa Ganguly, Mike Feldman, Brendan Kelly, Ronald G. Collman and Frederic Bushan | Bushman Lab - University of Pennsylvania | Hospital of the University of Pennsylvania Molecular Pathology Lab | 2021-04-10 | EPI_ISL_2597536 | **hCoV-19/USA/PA-VSP2225/2021** |
| Khailany,R.A., Rahman,M.O., Ozaslan,M. | GENETICS DEPARTMENT, ZHEEN INTERNATIONAL HOSPITAL | GENETICS DEPARTMENT, ZHEEN INTERNATIONAL HOSPITAL | 2021-03-09 | EPI_ISL_2153105 | **hCoV-19/Iraq/Erbil-1/2021** |
| Drechsel, Oliver | IMS-10146-CVDP-1204805D-6911-4D4C-86D5-02091960ED49 | Limbach - MVZ Humangenetik Ulm | 2021-03-16 | EPI_ISL_1350542 | **hCoV-19/Germany/BW-RKI-I-043354/2021** |
| Dr. Varsha Potdar | NIV Influenza | ICMR-National Institute of Virology - INSACOG | 2021-01-27 | EPI_ISL_1703962 | **hCoV-19/India/PB-ICMR-NIV-INSACOG-GSEQ-588/2021** |
| PHE Covid Sequencing Team | COVID-19 Genomics UK (COG-UK) Consortium | Respiratory Virus Unit, Microbiology Services Colindale, Public Health England | 2021-01-01 | EPI_ISL_2737121 | **hCoV-19/England/PHEC-159966/2021** |
| Sadri,N., Alouani,D., Song,X. | UHTL, University Hospitals | UHTL, University Hospitals | 2021-06-07 | EPI_ISL_2661529 | **hCoV-19/USA/OH-UHTL-992/2021** |
