# Supplementary material for: High prevalence of an alpha variant lineage with a premature stop codon in ORF7a in Iraq, winter 2020–2021
Source: PLoS One. 2022 May 26;17(5):e0267295. doi: 10.1371/journal.pone.0267295 (PMC9135184; doi:10.1371/journal.pone.0267295)
Supplement: Attachment — Submitted filename: Response to Editor.docx [file pone.0267295.s005.docx]

# **Response to Decision Letter and Reviewer Comments**

# **Manuscript number:** PONE-D-21-36713

# **Title:** High prevalence of an alpha variant lineage with a premature stop codon in ORF7a in Iraq, winter 2020-2021

# **Reviewers' Comments:**

# The sample size is not statistically significant. At least 500 sample needs to be analyzed before inferring something.

# Patients were selected just on the basis of viral titre (a high copy number of the virus (Ct values 110 <24)), the questions is were these patients immune competent before they caught infection? Do these patients have no comorbidity? The manuscript lacks information about clinical details of subjects.

# Those who died, did have any other disease that after infection might have resulted in death? Because the major portion of enrolled patients had mild to moderate symptoms in spite of high copy number of the virus.

# **Response:**

Dear Editor

Thank you very much for your valuable review. Here is a point-by-point response to the editor's comments.

As mentioned in response to the first and second reviewers, we agree that this number of genome sequences is not enough to cover the COVID-19 molecular epidemiology of the entire country. However, due to the limited sequencing capacity, there is currently very little information on the SARS-CoV-2 variants circulating in Iraq. Given that the global availability of SARS-CoV-2 genomic surveillance is extremely biased, we believe that covering this gap in mid-and low-income countries is highly valuable, and, albeit imperfect, the current study provides insight into the strains circulated during the second wave of COVID-19 in Iraq.

We have now more clearly addressed the limitations of the study in lines 382–386.

"The present study has a limited sample size, which may not represent the complete genomic diversity of SARS-CoV-2 in Iraq. It should be noted that the detected genomes may represent only locally distributed viral variations during the second epidemic wave, and the total diversity of SARS-CoV-2 circulating in Iraq is most likely significantly higher".

Regarding clinical details, we have now added information on comorbidities to Table S3. As mentioned earlier, the sample size is too small to correlate any variables with the disease outcome or clinical manifestations, and therefore, we are reluctant to speculate on these in the manuscript. Instead, we focus on genomic characterization and in silico assessment of the potential phenotypic effects of the observed amino acid substitutions.
